# Control fast or control smart: When should invading pathogens be controlled?

**DOI:** 10.1371/journal.pcbi.1006014

**Published:** 2018-02-16

**Authors:** Robin N. Thompson, Christopher A. Gilligan, Nik J. Cunniffe

**Affiliations:** 1 Department of Plant Sciences, University of Cambridge, Cambridge CB2 3EA, United Kingdom; 2 Department of Zoology, University of Oxford, Oxford OX1 3PS, United Kingdom; 3 Mathematical Institute, University of Oxford, Radcliffe Observatory Quarter, Oxford OX2 6GG, United Kingdom; 4 Christ Church, University of Oxford, Oxford OX1 1DP, United Kingdom; University of California, Los Angeles, UNITED STATES

## Abstract

The intuitive response to an invading pathogen is to start disease management as rapidly as possible, since this would be expected to minimise the future impacts of disease. However, since more spread data become available as an outbreak unfolds, processes underpinning pathogen transmission can almost always be characterised more precisely later in epidemics. This allows the future progression of any outbreak to be forecast more accurately, and so enables control interventions to be targeted more precisely. There is also the chance that the outbreak might die out without any intervention whatsoever, making prophylactic control unnecessary. Optimal decision-making involves continuously balancing these potential benefits of waiting against the possible costs of further spread. We introduce a generic, extensible data-driven algorithm based on parameter estimation and outbreak simulation for making decisions in real-time concerning when and how to control an invading pathogen. The Control Smart Algorithm (CSA) resolves the trade-off between the competing advantages of controlling as soon as possible and controlling later when more information has become available. We show–using a generic mathematical model representing the transmission of a pathogen of agricultural animals or plants through a population of farms or fields–how the CSA allows the timing and level of deployment of vaccination or chemical control to be optimised. In particular, the algorithm outperforms simpler strategies such as intervening when the outbreak size reaches a pre-specified threshold, or controlling when the outbreak has persisted for a threshold length of time. This remains the case even if the simpler methods are fully optimised in advance. Our work highlights the potential benefits of giving careful consideration to the question of when to start disease management during emerging outbreaks, and provides a concrete framework to allow policy-makers to make this decision.

## Author summary

Infectious disease outbreaks in human, animal and plant populations can have devastating consequences. Where and how much control should be deployed are difficult questions to answer. However, mathematical modelling is increasingly used by policy-makers to underpin these–sometimes contentious–decisions. When to manage disease is often viewed as more straightforward. Surely interventions should be introduced quickly, in an attempt to contain the outbreak as soon as possible? In practice, there are a number of unavoidable uncertainties at the beginning of any outbreak, such as whether or not the initial cases will lead to a major epidemic, as well as uncertainty about the exact transmissibility of the pathogen. We show that waiting to resolve these uncertainties before starting interventions can lead to less costly outbreaks, even though waiting means there might then be a larger outbreak to control. We have developed a novel algorithm that can be used in real-time during emerging outbreaks to decide when and how to introduce control. While for human diseases it might be controversial for policy-makers to recommend delaying interventions, for pathogens of plants or animals such a strategy will often be optimal and ethically justifiable. Our work shows how this approach might be implemented in practice.

## Introduction

Effective management of infectious disease remains a significant challenge [1–3]. Mathematical modelling is a powerful tool for integrating over uncertainty in predicting the future spread of outbreaks [4,5], and provides a rational methodology for assessing the likely performance of proposed interventions. Policy-makers can then base decisions concerning when, where and how to control disease outbreaks on quantitative scientific evidence [6–9].

Where and how epidemics should be managed has received significant attention, and a range of complex, often spatially-heterogeneous, control strategies have been proposed. These often target individuals at high risk by virtue of location [10–13] or contact patterns [14,15]. The question of when to introduce interventions appears much simpler. The difficulty of controlling an outbreak depends upon its size, and is expected to be easier and/or less expensive if treatment starts when the outbreak is small [9,16]. Early action also potentially avoids the feedback by which the net growth rate of an epidemic increases as the outbreak gets larger [17]. Intuition therefore suggests that control should be performed as soon as possible, a result echoed in a number of modelling studies [12,18–20].

However, intuition is seldom infallible, and optimising the timing of disease management can be more nuanced. Early control is only guaranteed to be effective if there is absolutely no uncertainty surrounding the future spread of disease. Demographic stochasticity can lead to an outbreak dying out without any management before a major epidemic occurs [21,22]. For certain plant and animal diseases it might therefore be optimal simply to wait and see whether or not the outbreak takes off, potentially avoiding wasted prophylactic control [23]. Withholding or delaying treatment creates ethical problems over each individual’s right to treatment, particularly for human diseases. Nevertheless for emerging outbreaks of humans as well as of crops and livestock, control interventions can almost always be targeted more effectively later in epidemics, when more is known about the dynamics of disease spread. The optimal management strategy is typically conditioned on the values of parameters controlling pathogen transmission [24,25], often quite sensitively [13,26,27]. Epidemiological parameters can be characterised increasingly accurately as an outbreak progresses, since more data are then available [28–30]. This sets up a trade-off, in which the additional accuracy with which control could be targeted if management was to be delayed could potentially outweigh the increased cost of controlling a larger epidemic. This trade-off raises a question: when precisely is it optimal to treat?

To work in a concrete but still general setting, we consider a large-scale epidemic affecting agricultural animals, which we model using a stochastic, compartmental model of pathogen transmission in which the host units represent individual farms. This broad class of model–or elaborations to include a spatial component or other additional population heterogeneities–is routinely used for a number of animal diseases including classical swine fever [31,32] and bovine tuberculosis [11,33], as well as for pathogens of plants [3,9,34] and humans [7,35,36]. Estimated values of transmission parameters become more precise as the outbreak unfolds and more data to fit the model become available. Control involves prophylactic vaccination of all animals on a fraction of susceptible farms. Vaccination protects farms for the duration of the outbreak, and, for simplicity, we assume vaccination can be done only once. The overall aim is to minimise the total cost of the outbreak, which consists of the cost of herds lost to disease, together with a smaller cost assigned to each farm on which animals are vaccinated.

While we refer to a disease of livestock and a control strategy based on vaccination here, this is only to provide a simple scenario that is straightforward to describe and to visualise. The main concept that we investigate–i.e. whether or not an initial period of observation and learning might be a sensible consideration in an outbreak response setting–is likely in fact to be applicable to a large number of pathogens and controls. We introduce the extensible Control Smart Algorithm (CSA) to determine when and how to control in real-time. The CSA integrates disease spread data, parameter estimation and outbreak simulation to determine whether to control now, or whether instead management should be delayed to allow the policy-maker to learn more about transmission and the likely severity of the outbreak. Crucially, the CSA takes account of how rapidly any imprecision is likely to be resolved, comparing this timescale with the time at which the epidemic is expected to become too large to control effectively. The CSA is used repeatedly at successive times at which control could potentially be introduced, leading to a sequence of decisions on whether or not to intervene, as well as a specification of the control to perform when management is finally to be attempted.

We address the following questions.

1. Should disease management always be introduced immediately, or can it instead be optimal to introduce control after the start of the outbreak?
2. If delaying management can be optimal, is this driven by increasing precision in estimates of epidemiological parameters, by the possibility of the outbreak dying out, or by some combination of these factors?
3. How can decisions on when to implement control be made in real-time during an emerging outbreak?
4. To what extent does our dynamic approach to deciding when to begin treatment outperform simpler strategies that can be optimised in advance of the outbreak?

## Results

### Can delaying disease management be sensible?

**Fig 1:**
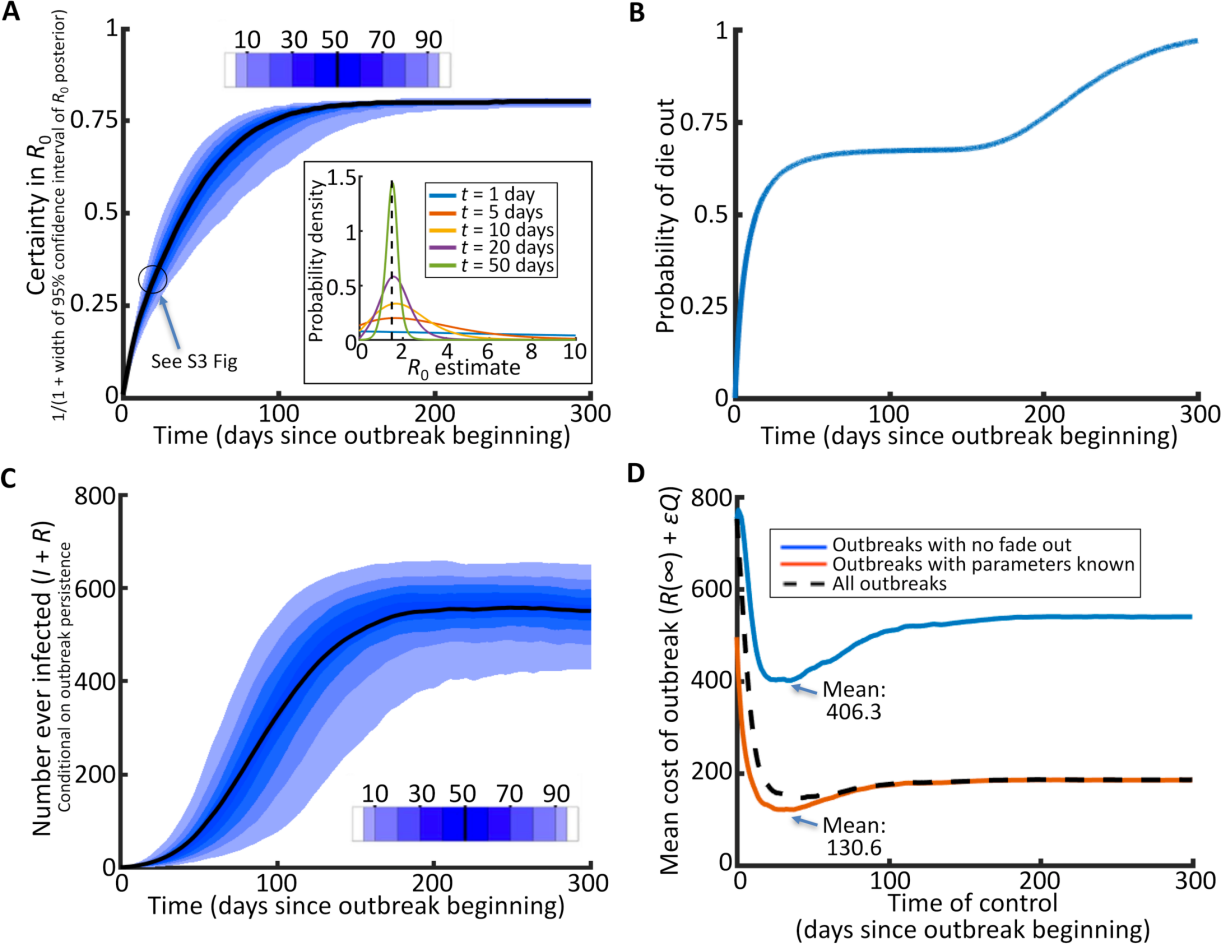
Trade-off between information gain and outbreak size. A. Conditional on persistence, estimates of *R*_0_ improve as the outbreak progresses (95% confidence interval (blue), median (black)). The inset indicates how the posterior density for *R*_0_ changes when estimation occurs at successive times in the outbreak; B. As time goes on, the chance of the outbreak dying out increases, so control is potentially unnecessary; C. An ensemble of outbreaks showing how the numbers of farms ever infected increases over time (95% confidence interval (blue), median (black)). Waiting before deploying control potentially leads to a larger epidemic, making the pathogen harder to control; D. The optimal time to control an invading outbreak depends on the trade-offs amongst the three components: the degree of (un)certainty in *R*_0_; probability of fade out; and the impact of the epidemic (A, B, and C respectively, above). The optimal control time is the time corresponding to the minimum expected outbreak cost. The cost function comprises of the number of farms infected (*R*(∞)), the cost of vaccination per farm (ε) and the number of farms vaccinated (*Q*). Panel D shows: outbreaks that persist for at least 50 days and parameters are estimated from the transmission data (blue); outbreaks in which parameters are known and do not require estimation (red); and all outbreaks, including those which quickly die out, with parameters requiring estimation from the transmission data (black dotted). The estimation procedure we use for panels A and D is described in Methods and S5 Text, and a schematic describing how the certainty in *R*_0_ is then calculated in A is in S3 Fig. In panel D, control is optimised using the CAOA (S2 Fig; S1 Algorithm). Each panel is based on 100,000 underlying simulated datasets (with 100,000 datasets for each of the blue, red and black dotted lines in D) generated using the stochastic SIR model in a population of size *N* = 1,000 with between-farm *R*_0_ = 1.5, μ = 0.1 per day, ε = 0.8, and initial number of infected farms *I*(0) = 1.

At any time during an emerging outbreak, the optimal number of farms on which to deploy vaccination depends on the current outbreak size and the values of parameters governing transmission. We showed how this optimal number can be estimated using the stochastic SIR model and how this number depends on the model parameters (S1 Text; S1 Fig). If a policy-maker is presented with an outbreak for which the infection statuses of every farm and the values of transmission parameters are all known, rather than simply the infection statuses as we assumed here, then optimising control is very straightforward. However, the estimated values of parameters change as an epidemic unfolds, and during this time the current outbreak size also changes. To understand time-dependence of these factors that drive the optimal amount of control to deploy, we simulated a very large number of outbreaks, quantifying temporal changes in the precision of parameter estimates (Fig 1A), the probability of the outbreak dying out (Fig 1B), and the size of the outbreak (Fig 1C). We then tested the effect of introducing control on each day in these simulated epidemics, using parameter estimates from data up to and including the day in question to determine the most efficient management at that time. To optimise the extent of control deployment, we used the Control Amount Optimisation Algorithm–CAOA, shown in S1 Algorithm and S2 Fig. The CAOA averages over uncertainty in the effect of management as well as uncertainty in the transmission parameter values via repeated forward simulation.

The average cost over all simulated outbreaks was minimised with control deployed after 40 days (dotted black line in Fig 1D). Since delaying control was initially optimal, the trade-off between the additional accuracy with which control can be targeted and the increased size of the epidemic led to a non-zero delay before intervening became optimal. To separate the effects of increasingly precise parameter estimates from those caused by outbreaks potentially dying out naturally, we also examined only the subset of simulated outbreaks that persisted for more than 50 days in the absence of control (blue line in Fig 1D). The response of the average outbreak cost to time of treatment again had a minimum, here at *t* = 28 days, meaning that uncertainty in parameters alone favoured delayed control. When uncertainty about model parameters was removed instead, so that the exact value *R*_0_ = 1.5 was used to optimise the amount of control deployed, we found that it was still optimal to wait before implementing control (the minimum was now at *t* = 30 days, red line in Fig 1D). The chance of natural disease fade-out therefore also suggested an optimal strategy of waiting before starting treatment.

### How can decisions be made in real-time during an emerging outbreak?

**Fig 2:**
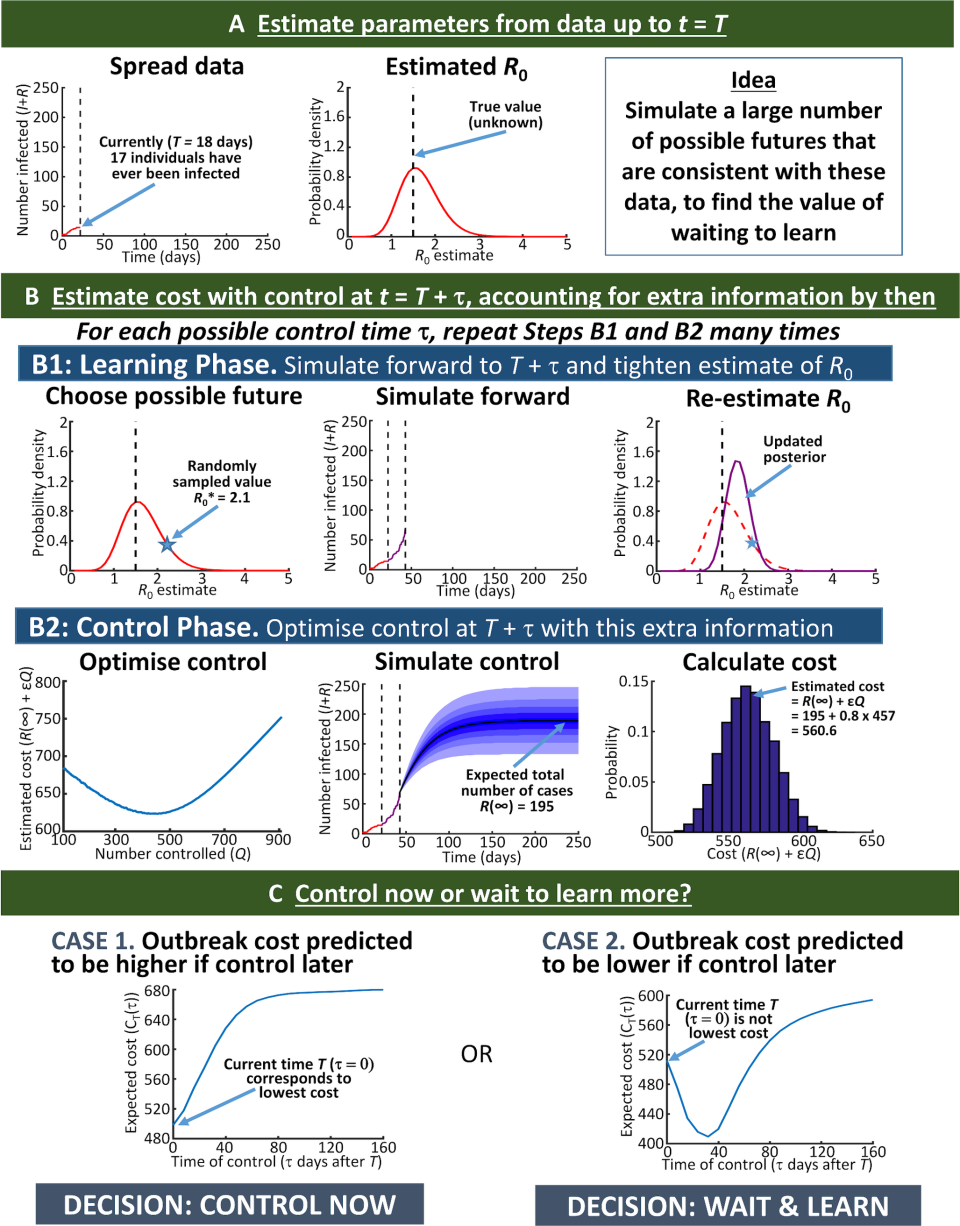
How can a decision be made as to whether to control at the current time or wait to control in future? Schematic describing the Control Smart Algorithm (CSA; S2 Algorithm).

The optimal time to control any individual ongoing outbreak will usually differ from that dictated by the average behaviour over a large ensemble of outbreaks. We therefore developed the Control Smart Algorithm (CSA), a dynamic approach for deciding when to implement control in real-time during a single ongoing outbreak (see Fig 2 and Methods).

**Fig 3:**
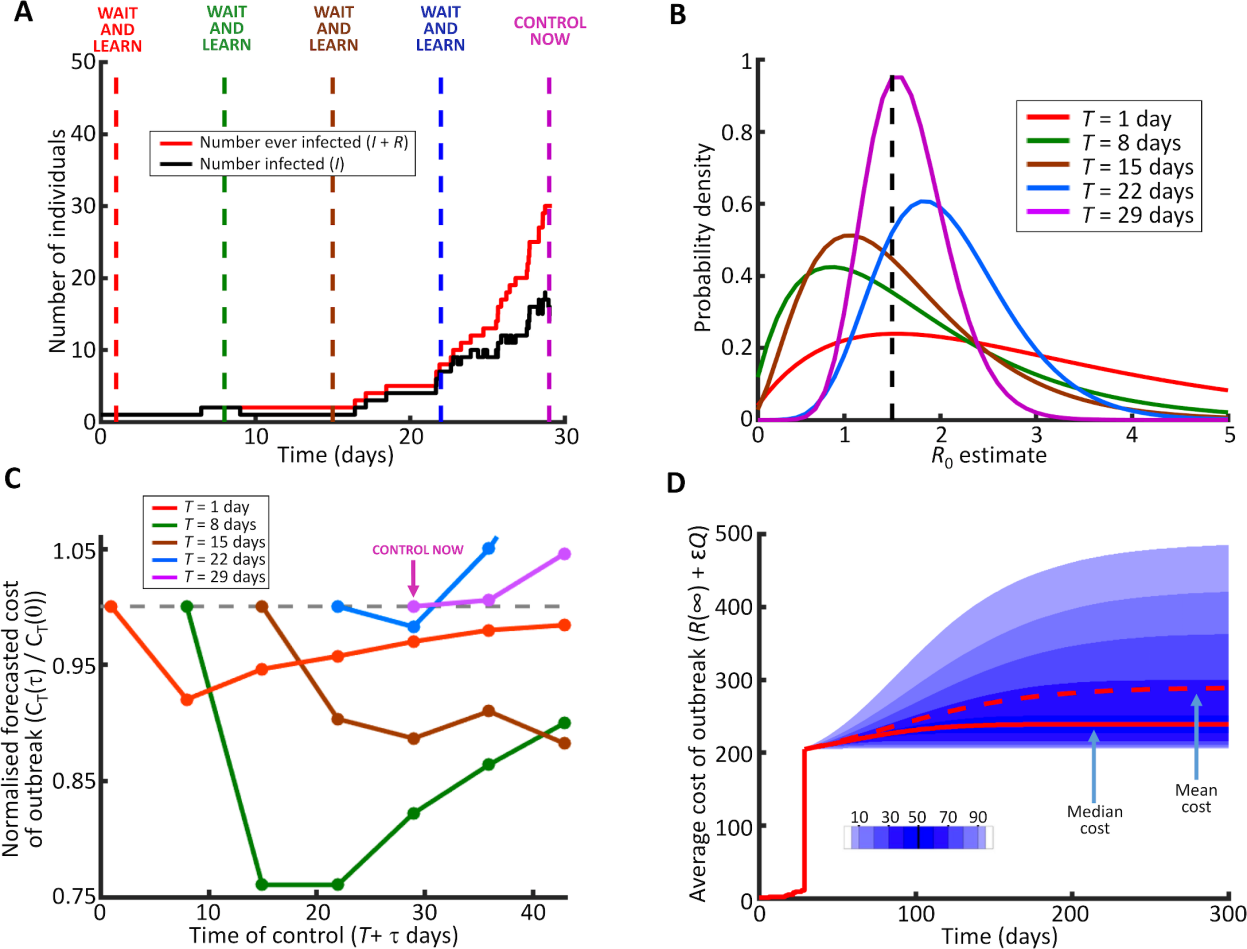
Using the CSA to decide when to introduce control. A. A simulated dataset, with weekly possible decision times starting when *T* = 1 day; B. Posterior estimates of *R*_0_, which become more precise as more outbreak data are available; C. Forecast cost of the outbreak with control at each time in the future, for each control time. The first time at which the current time is predicted to be the best to deploy control is T* = 29 days; D. The true distribution of possible costs of the outbreak, when control is introduced at T* = 29 days according to the CSA. Parameter values: *N* = 1,000 with *R*_0_ = 1.5, μ = 0.1 per day, ε = 0.8. In C, 100,000 forward simulations are generated for each control time in future to estimate the expected cost of the outbreak. In D, 100,000 forward simulations are run from T* = 29 days to show the true expected future behaviour of the outbreak. In D, the true value of the basic reproduction number (*R*_0_ = 1.5) is used in forward simulations to generate the true expected cost of the outbreak, whereas in C the estimated costs of the outbreak are generated using the posterior parameter values estimated at the relevant decision time *T*.

We first showed how the CSA performs on a synthetic dataset from a single simulated outbreak, allowing for decisions concerning whether or not to introduce control on a weekly basis (Fig 3). For the example epidemic that we initially considered, there were a number of decisions to wait, followed by the decision to control after *T* = 29 days (Fig 3A). The estimated values of transmission parameters became increasingly precise during the waiting phase (Fig 3B). Control was introduced at the first possible intervention time at which the expected cost of the outbreak when controlling at the current time was less than the equivalent expected cost at all possible future control times (i.e. the first time at which the curve in Fig 3C was monotonic increasing). Of course even after control was introduced, demographic stochasticity meant that a range of outcomes remained possible, even though *R*_0_ was then fixed at the true value (Fig 3D).

**Fig 4:**
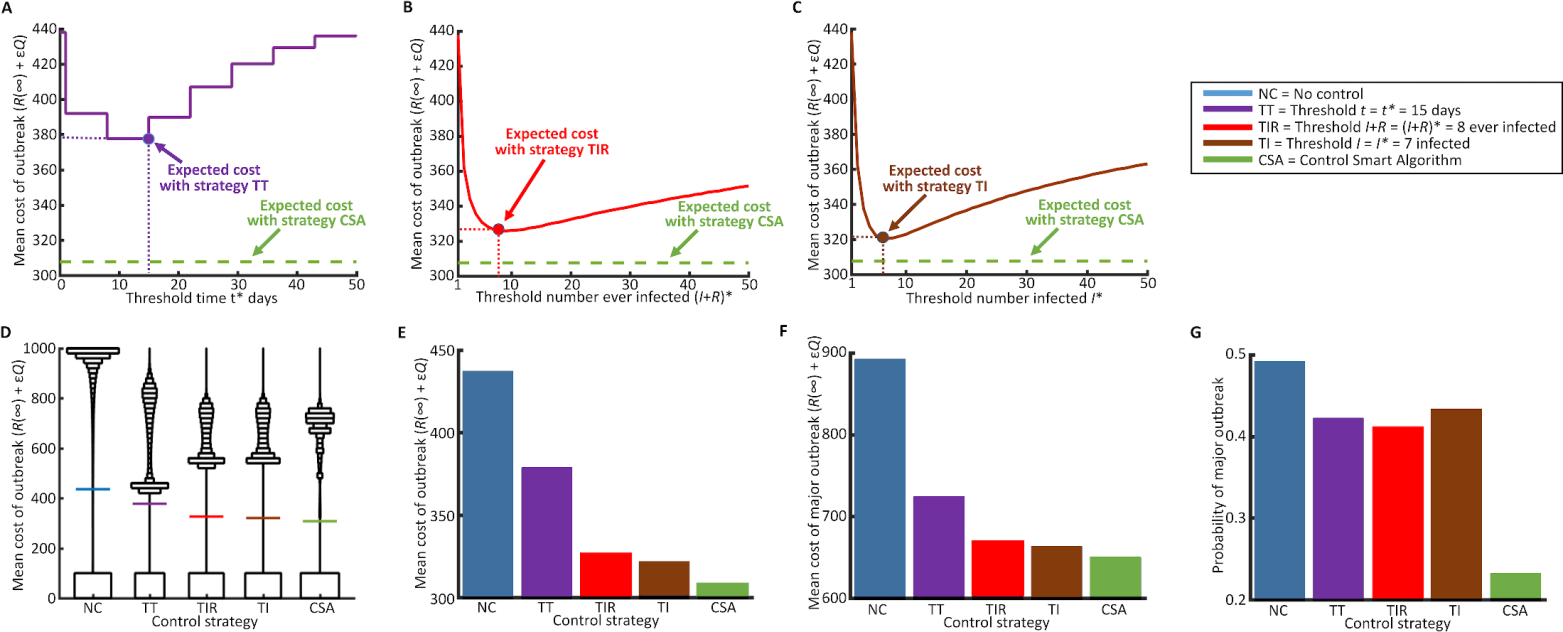
Using the CSA reduces the expected cost of outbreaks and the probability that there will be a major epidemic. A. The expected cost of outbreaks with control according to strategy TT (purple), compared with the expected cost using the CSA (green); B. The expected cost of outbreaks with control according to strategy TIR (red), compared with the expected cost using the CSA (green); C. The expected cost of outbreaks with control according to strategy TI (brown), compared with the expected cost using the CSA (green); D. The distributions (see explanation below) of outbreak costs under each control strategy. Mean values are indicated by coloured horizontal lines; E. Mean cost of outbreaks with control according to the optimised threshold-based strategies described in Methods (*cf*. horizontal lines in panel D); F. Mean cost of major outbreaks (which here we define as outbreaks in which the number of individuals ever infected exceeds 20); G. Probability of a major outbreak under each control strategy. In D-G, the values of the relevant thresholds corresponding to lowest costs are used. These correspond with the optimal thresholds and control amounts that could be predicted in advance of the outbreak. In D, the areas of the bars are proportional to the numbers of outbreaks within the corresponding cost values on the y-axis. Due to the large number of outbreaks that have very low costs, costs in the range 0–100 are combined. Note that the areas of the lowest bars in D do not correspond to the values in G, since a "major outbreak" in F and G is defined by the number of individuals infected during the outbreak rather than the total cost (which includes both individuals infected and the cost of vaccination). Parameter values: *N* = 1,000 farms, μ = 0.1 per day, ε = 0.8. In A-C, 1,000,000 simulations are run per threshold value and in D-G, 1,000,000 simulations are run per distribution/bar. Underlying simulated datasets are generated with values of *R*_0_ chosen uniformly at random from the interval (0,5). The decision of whether or not to introduce control is made on a weekly basis, starting at *T* = 1 day. For the threshold strategies, control is introduced at the first possible intervention time after the threshold condition is met.

By repeating this analysis for a large number of different simulated epidemics for a range of values of *R*_0_, we compared the performance of the CSA with those of simpler methods for deciding when and how to control (Fig 4). In particular, we tested the CSA against alternative strategies in which control was deployed as soon as thresholds in outbreak severity or duration were reached [37]. By optimising the threshold-based methods in advance (S2 Text), we identified the following thresholds: duration of the epidemic (TT strategy; *t* = t* = 15 days; Fig 4A); number of farms that have ever been infected (TIR strategy; (*I*+*R*) = (*I*+*R*)* = 8; Fig 4B); and number of farms currently infected (TI strategy; *I* = I* = 7; Fig 4C). The CSA reduced the mean cost averaged across all outbreaks compared with these simpler methods (Fig 4D–4E). Although the single goal of the CSA was to minimise the mean outbreak cost, using this method also reduced the cost of those outbreaks that did invade (Fig 4F) and the probability of a major epidemic (Fig 4G).

Our objective here was to introduce and test the CSA in the simplest possible setting, in which the infection rate was the only parameter requiring estimation from disease spread data and in which there were no constraints on control deployment. However, we additionally verified that the improved performance of the CSA in comparison to the simpler methods of control was robust to informed prior knowledge of the parameters (S4 Fig), estimation of multiple parameters (S5 Fig) and logistic constraints in control (S6 Fig). We also tested robustness to the accuracy of prior knowledge concerning epidemiological parameters (S7 Fig), as well as to systematic bias in this prior knowledge (S8 and S9 Figs). These elaborations to the CSA are described in S3 Text. For computational tractability given the extremely large number of simulated outbreak datasets we considered in these extensive sensitivity analyses, we ran the underlying simulations in a smaller population of *N* = 100 host farms. In all cases the CSA outperformed the other simpler threshold-based strategies for determining when and how to control.

## Discussion

Delaying management to allow more information to be accrued has been proposed only rarely for infectious diseases [38,39], despite work focusing on a similar idea for managing invasive species [40–43]. Here we have shown–using a generic stochastic model capturing the key features of an emerging disease outbreak–that it can be beneficial to delay control of potential epidemics (Fig 1). This allows outbreaks that would die out naturally to do so at no additional cost. It also allows disease spread parameters to be estimated more accurately, which in turn allows management to be targeted more precisely. Having verified that deferring control can be optimal via a *post-hoc* analysis in which the costs of numerous simulated outbreaks were averaged, we introduced the Control Smart Algorithm (CSA, Fig 2). This algorithm could be extended for use in real-time to inform decision-making concerning when to control an ongoing outbreak. It makes use of stochastic compartmental models and rigorous parameter estimation techniques of the type routinely employed for real-time infectious disease epidemic forecasting.

The CSA uses forward simulation to optimise the timing and amount of control to introduce. The algorithm scans over possible control interventions, and all possible values of the parameters governing pathogen transmission. By averaging according to entire posterior distributions for the values of these parameters, a pre-specified objective function derived from the distribution of costs of forward simulations can be minimised. Here, we used the mean cost of outbreaks as this objective function, and the expected cost of the outbreak with control now and the equivalent expected cost with control in future were calculated exactly. For this reason, the expected cost of outbreaks with control according to the CSA as presented here will always be lower than with any other possible control strategy, given a posterior estimate of the parameters. This is the case even if these other strategies are fully optimised in advance of the outbreak (Fig 4).

Of course, if the posteriors of the parameters are incorrect, then the CSA will be badly informed, and so in that case it might be possible for other control strategies to outperform the CSA. In reality there is often prior information that leads to systematic bias in the decision-making, for example observations of previous epidemics of the same pathogen but in different environments. However, a strategy of waiting before deploying control has the additional benefit that evidence to overcome this initial bias can be accrued. We showed the robustness of our method to the choice of prior (S4–S9 Figs), including situations with systematic biases in the decision-making (S8 and S9 Figs).

The benefit of waiting to learn about disease spread, and the rate at which this uncertainty can be resolved compared to the speed of the outbreak, are likely to impact on whether or not waiting before deploying control is a sensible strategy. As we have described, the CSA performs this assessment rigorously given outbreak data from up to the current time and priors on the values of transmission parameters. In cases where uncertainty can only be reduced slowly throughout an outbreak, then early control will be advantageous. Early control will avoid the outbreak size becoming large before the most efficient control strategy can be deduced. The recommendation of the CSA in this case would most likely be to control immediately.

The concept presented here, that waiting to introduce management might outperform a strategy of controlling immediately, was inspired by the similar idea of “wait and see” approaches to invasive species management [40,41,44,45]. In the pest management setting, it has been shown that it can be most effective to survey sites intensively to learn where the pest is most likely to be found before introducing a mixed strategy of searching and removal [40]. The idea of investing in collecting data has also been considered for protecting habitat critical for species survival, by asking when interventions should be introduced to avoid an unacceptable risk of species extinction [43]. The optimal times at which to change management strategies have also been considered in the endangered species management setting [42,46]. Of course, while delaying control can be effective for invasive species, waiting too long is also ineffective [47,48]. We have shown here that this principle also applies to controlling invading pathogens.

While studies about when to introduce interventions are rare for infectious diseases, certain outbreaks are thought to have perhaps been over-controlled. For example, costly widespread management interventions were enacted in 2009 to counter the perceived potential pandemic of H1N1 influenza [49]. However, the outbreak was far less serious than experts had forecast, leading to speculation that control deployment was disproportionate to the level of threat [50,51]. There are also a number of outbreaks, such as the 2007 United Kingdom foot-and-mouth outbreak, in which disease fade out has been used to justify the preceding aggressive interventions [52]. However, despite the small size of these outbreaks, control can be expensive [53], and outbreaks might be minor in the absence of attempted eradication strategies [22]. Immediate intervention might therefore be unnecessary and an avoidable cost.

Such high-profile examples of the potentially sub-optimal effects of very early management have motivated the invention of various metrics for guiding whether or not to act. One example is the first 14 days incidence, FFI, which has been proposed by Hutber *et al*. [54] as a parameter for informing whether or not to begin emergency vaccination campaigns. The closely-related first 14 days spatial spread parameter, FFS, was developed subsequently [55]. Outbreak simulations indicate that there is a strong positive correlation between FFI/FFS and variables indicative of outbreak severity such as final size and outbreak duration [56,57]. Further studies have strengthened the evidence that simple metrics available early in outbreaks are able to predict final epidemic characteristics [56]. Such metrics can therefore form the basis of decision-making tools that present important information about the possible impacts of control interventions in a simplified format for policy-makers [57].

Other modelling analyses that seek to avoid unnecessary early control are based on real options, which attempt to understand and value potential benefits of waiting before introducing disease control [58]. Epidemiological applications include the timing of investment in antiviral drug stockpiles [59] and control of plant disease outbreaks [38,60]. The real options approach has illustrated the principle that when there is uncertainty in future pathogen/pest progression through the host population, combined with the sunk costs of control measures, there can be value in waiting instead of deploying control immediately [61–63]. How long the decision-maker should wait depends on a number of factors. These include the level of uncertainty in the future spread of the outbreak [60], whether or not proposed interventions are reversible [59] and policy-makers’ flexibility in choosing the timing of interventions [38]. However, previous studies using real options have not explicitly considered the fact that as the epidemic progresses and more data are collected parameter estimates become more precise. As we have demonstrated here, uncertainty in transmission parameter values is an important factor in determining the optimal timing for the introduction of control measures.

Resolving other types of uncertainty might also be important in practice. So-called “Value of Information” analyses [64–67] have been proposed to estimate the costs associated with uncertainty. For example, the “Expected Value of Partial Perfect Information” was recently used in the context of foot-and-mouth disease to estimate the costs associated with uncertainty about a number of factors including vaccine efficacy and the time delay to immunity after vaccination [27]. Value of Information analyses can inform adaptive management strategies, in which disease controls are changed over time as more information becomes available and model uncertainty is resolved [68]. An adaptive management strategy has also been proposed to show how dynamically changing vaccination strategies can reduce the costs of epidemics [69]. However, the question of when to introduce control in the first place has not been considered in that type of study.

Much closer to the current study is a dynamic methodology for deciding when to control an epidemic that was introduced in the context of influenza management by Ludkovski and Niemi [70]. In that approach, a set of outbreak statistics (observed states of the system and estimates of disease spread parameters) are chosen to characterise the current severity of the outbreak and the current level of predictability of future spread. Policy maps are then created using regression Monte Carlo, which specify values of these outbreak statistics for which control should be introduced immediately [70]. For efficient use, the policy maps must, however, be generated in advance of the outbreak. In contrast, the CSA does not require outbreak statistics to be chosen in advance, but instead uses all the information available to policy-makers during an emerging outbreak. The CSA also only simulates forwards from the current observed state of the system, thereby avoiding additional computational costs of exploring future behaviour that is no longer possible given the progress of the outbreak so far.

We framed our analysis in terms of stochastic SIR dynamics, which we adopted here to represent only the necessary features of an outbreak for which there is uncertainty about transmission parameter values and future spread [24,71]. We used synthetic data generated using model simulations to illustrate the concept that the optimal time to introduce control might not be immediately, and also to test our results for a very large number of different outbreaks. In practice for any single particular outbreak, it would be necessary to use a detailed model more finely-tuned to the particular host-pathogen system in question. However, since our results and the CSA are largely based on forward simulation, in principle such alterations could be made without any significant methodological change. Indeed the elaborations to the CSA that we presented in S4–S9 Figs showed how additional detail can be included in the underlying methodology in simple cases. More exhaustive investigations exploring systematically how variations in model complexity–or alterations to the elaborations that we considered–impact on the performance of the CSA relative to the simpler methods for deciding when and how to control are left as future work. For example, an analysis analogous to S6 Fig in which the limit to the number of farms that can be controlled per day can take a range of values could be considered.

The CSA could also be adapted for use with more complex epidemiological data and methods of parameter inference. Here we assumed that all epidemiological transitions made by all hosts were fully recorded to facilitate easy parameter estimation, but this is not necessary; the only requirement is that posterior estimates of epidemiological parameters can be generated. Other sources of uncertainty could also potentially be accounted for in the CSA, for example uncertainty in the number of currently infected host units arising either because of underreporting [72] or presymptomatic/asymptomatic infection [22], and uncertainty in which model out of a suite of plausible compartmental models most accurately represents disease spread [73]. However, we defer systematic testing of the performance of the CSA with these additional sources of uncertainty to future work.

Other simplifications underlie our work. Control made susceptible farms immune to the disease for the remainder of the outbreak. Although simple, such behaviour is consistent with a range of pre-emptive controls, including vaccination, preventative culling/thinning and application of protectant fungicide or pesticide. However, other controls could instead have been adopted, singly or in combination. Complexities such as resource-driven limitations to the capacity for treatment [10] or delays between decision-making and control deployment [12] could also have been included. To demonstrate how the CSA can be extended to account for considerations like these, we built S6 Fig in which a limit to vaccination deployment was included in the analysis in a simple way, showing at least in principle that the CSA is robust to this additional complexity. Our metric for assessing the cost of outbreaks was also rather simple. Elaborations could include a discount rate [74], more complex notions of costs of infections and control [75], and multiple objectives [76,77].

As a proof of concept, we focused entirely on minimising the mean cost of an outbreak in this manuscript. This choice could potentially lead to outbreak features that might be undesirable for policy-makers. For example, in Fig 4D it can be seen that, despite the CSA having the minimum mean cost, on those occasions where costly outbreaks do occur the distribution of costs under the CSA is “top heavy” compared with the other strategies. Practical implementation of the CSA for disease outbreak management would require careful consideration of precisely which objective function to minimise. The mean cost of forward simulations may not be the preferred choice, since this quantity can be influenced by simulations with extremely high or low costs. For real outbreak response scenarios, policy-makers might therefore prefer to optimise quantities such as the median forecasted outbreak cost, or even to focus on an objective function taking a weighted average of a number of different metrics. Our underlying method could also handle other levels of risk aversion, for example by considering achieving optimal performance for different percentiles in the distribution of outbreak costs [12]. Since control and its cost only enter the CSA via forward simulation of the disease spread model, in principle interventions and cost functions of arbitrary complexity would be admissible.

While additional features could be introduced into the underlying methodology that we presented, we note that efficient management is often beset with technical and operational challenges [4,78,79]. Identifying which factors to include in the model of spread and the cost function is an important problem, particularly for emerging outbreaks of novel pathogens during which there are numerous uncertainties. It must also be decided which interventions are likely to be feasible before the CSA can be used. Even if possible control strategies and factors that affect the impact of the outbreak can be identified, the optimal level of detail to incorporate in the modelling study is a fine balancing act. Communication between modellers and policy-makers is essential [79].

However, while extensions to the CSA are possible, our goal was to introduce the algorithm in a relatively simple setting. We therefore only considered a single management intervention, and assumed that control cannot be changed later in the outbreak. This type of restriction might well be in place in practice, since governments might only be able to set their policy once, or at most change it a limited number of times. Extending the CSA to account for multiple changes in policy would be interesting, although computationally challenging to trial on large numbers of simulated outbreaks. Even with a single time of control introduction, the CSA is computationally expensive to test on many simulated datasets due to the need to run forward simulations with control both at different times in the future and with different amounts of control deployed. However, we note that in practice decisions are required for only one single outbreak (i.e. practical use would require only an analogy of Fig 3, not of Fig 4), greatly reducing computation times.

While the approach of waiting before deploying control when an outbreak is underway might pose an ethical dilemma for policy-makers, we have demonstrated the principle that delaying can minimise the expected cost. We have also introduced the CSA, a dynamic strategy for determining when to introduce management. As we have described, extensions could include considering multiple possible times at which interventions can be modified, additional complexity in epidemiological data and available controls, and applying the CSA with more complex models appropriate for outbreak data in a real outbreak response scenario. However, we have demonstrated that the underpinning question of whether or not to introduce control as quickly as possible should be carefully considered in future emerging outbreaks.

## Materials and methods

### Modelling disease spread

The deterministic SIR model describing the spread of disease in a population of *S*+*I*+*R = N* host farms is

$$\frac{dS}{dt}=-\beta SI,\;\frac{dI}{dt}=\beta SI-\mu I,\;\frac{dR}{dt}=\mu I,$$

in which β*I* is the per capita rate at which susceptible farms become infected, and μ is the rate at which farms are removed in the absence of control. The variables *S*, *I* and *R* represent the numbers of susceptible, infected and removed host farms in the system at time *t*. Our simulations use the analogous stochastic model (S4 Text) and are generated using the direct method version of Gillespie's stochastic simulation algorithm [80]. For the simulated datasets underlying Figs 1 and 3, we choose parameters such that the farm-level basic reproduction number is *R*_0_ = βN/μ = 1.5 (in particular *N* = 1,000, β = 0.00015 per day, μ = 0.1 per day). The variable *R*_0_ represents the average number of farms expected to be infected by a single infected farm if all other farms in the system are susceptible. To test the Control Smart Algorithm (CSA) more extensively, we later run simulations for *R*_0_ in the range 0–5 by varying β (Fig 4). We note that these values are within the range of estimated farm- or flock- level reproduction numbers for foot-and-mouth disease [81,82], avian influenza [83,84] and bovine tuberculosis [11], although detailed modelling of any particular disease is not the aim of our study.

### Modelling disease control

At a variable time, *T*, we consider introducing a control involving vaccination that completely removes a number of farms from the infection process for the duration of the outbreak. Although we consider vaccination specifically, a control focussed on treating healthy individuals is in keeping with other treatments of infectious diseases including removal of susceptible hosts and culling of hosts close to known infections [6,81].

The total cost of the outbreak is given by

Costofepidemic=R(∞)+εQ,

where *R*(∞) is the total number of farms lost to disease during the epidemic, *Q* is the total number of farms that are vaccinated and ε is the relative cost of control per farm compared to losing that farm to disease. This cost could represent a number of factors, including deploying the control itself [85,86], compensating farmers [87] and impacts on the rural economy and tourism [88].

### Parameter inference

We assume for simplicity in our main analyses that the infection rate between farms, β, is the only unknown outbreak parameter, and we assume that information on all epidemiological transitions made by all hosts is available. This allows us to generate easily a posterior distribution for the basic reproduction number, *R*_0_ (S5 Text). However, we note that in principle, any epidemiological data and/or method of parameter estimation could be integrated into the decision-making framework that we present. This includes extending the approach presented in the main text here to scenarios in which multiple parameters require estimation from disease spread data (S5 Fig) or in which there are informative priors concerning parameter values (S4–S9 Figs).

### Optimal amount of control when there is uncertainty

The amount of control to deploy if interventions are to be introduced immediately can be chosen by running forward simulations of the stochastic model with different amounts of control according to the Control Amount Optimisation Algorithm, CAOA (S2 Fig; S1 Algorithm). In each forward simulation, to assess the expected effect of control given the range of transmission parameter values consistent with data up until the current time, parameter values are sampled from the current posterior distribution. By repeating this process for each possible amount of control (i.e. numbers of susceptible individuals to vaccinate, *Q*), the expected cost for each possible amount of control is estimated. The optimal amount of control to use is that corresponding to the minimum expected cost.

### Control Smart Algorithm (CSA)

The CSA (Fig 2; S2 Algorithm) generates an estimate of the expected cost of an ongoing outbreak if control is introduced immediately (i.e. at *t* = *T*), and the expected costs if control is instead introduced at each possible time in future (i.e. at *t* = *T*+τ, a delay of τ days before introducing control). If the expected outbreak cost is lower with control deployed now rather than at any time in future, then the decision to control immediately should be taken. If not, then a decision of waiting to learn more and reassessing at the following possible decision time is recommended.

Here we present a more detailed description of how, at time *t = T*, an estimate of the cost of the outbreak with control at time *t = T*+τ is constructed. First, a posterior distribution for *R*_0_ is generated using disease spread data from the real ongoing outbreak up to current time *t* = *T*. This is the only time that real data are used in the CSA. Then, a value of *R*_0_ is sampled from this posterior and used in model simulations between *t* = *T* and *t* = *T*+τ. Control is then deployed in the simulation at time *T*+τ according to the CAOA (S1 Algorithm). However, the amount of control that is deployed in the simulation is calculated using the additional simulated information generated in the simulation between times *T* and *T*+τ. In this way the amount of control introduced in the simulation is the amount that would be introduced in reality, if the forward simulation was actually to occur. The simulation is then continued further from time *T*+τ to generate a single simulated outbreak cost. By repeating this for a large number of simulated outbreaks with different sampled values of *R*_0_, the estimated expected outbreak cost with control at time *T*+τ is generated.

An interesting but subtle feature of an optimal control strategy is that it does not necessarily involve performing the intervention that is predicted to be optimal if it is the only one deployed until the end of the outbreak. Instead ideal control depends on the intervention options that will be available in future. For example, the optimal control intervention at the current time will be different if the next opportunity to intervene is near in future or a long time away. Similarly, if control if irreversible, then it might be better to deploy only small amounts of control initially, and intervene more intensely later when improved parameter estimates provide a greater weight of evidence that heavy handed control is likely to be beneficial. A key feature of the CSA is that the optimal future control decisions are programmed into the forward simulations, allowing the best decision accounting for all possible future changes in intervention policy to be taken, rather than simply recommending the control that is likely to be best if unchanged throughout the rest of the outbreak.

In S6 Text we present tests showing that the CSA recovers the exact solution that can be derived using dynamic programming in the artificial case in which the values of all parameters are known (see also S10 and S11 Figs).

### Threshold-based control strategies

In comparing the CSA with threshold-based strategies to determine when to introduce control (Fig 4), we consider the following strategies.

1. NC—No control
2. TT—Control when outbreak has persisted for a threshold amount of time t*
3. TIR—Control when the number of farms ever infected reaches a threshold (*I*+*R*)*
4. TI—Control when the number of farms currently infected reaches a threshold I*
5. CSA—Control according to the CSA (Fig 2; S2 Algorithm)

For strategies 2, 3 and 4, the amount of control to introduce is optimised in advance of the outbreak (S2 Text).

## Supporting information

S1 Text Optimising the amount of control to deploy. (DOCX)

S2 Text Optimising the threshold-based control strategies. (DOCX)

S3 Text Elaborations to the Control Smart Algorithm (CSA). (DOCX)

S4 Text Simulations of the stochastic SIR model. (DOCX)

S5 Text Parameter inference. (DOCX)

S6 Text Testing the Control Smart Algorithm (CSA). (DOCX)

S1 Algorithm Control Amount Optimisation Algorithm (CAOA). (DOCX)

S2 Algorithm Control Smart Algorithm (CSA). (DOCX)

S3 Algorithm Simulation-based method for deciding whether or not to control at the current time (analogous to the CSA but with parameters known). (DOCX)

S1 Fig How does the cost of an outbreak vary depending on the amount of control that is introduced, and the model parameters? A. Cost due to control, εQ, for different costs of control ε; B. Expected cost due to individuals becoming infected during the outbreak, *R*(∞); C. Expected total cost of the outbreak for different costs of control ε, evaluated as the sum of A and B; D. Dependence of the expected cost of the outbreak on different initial numbers of infected individuals; E. Dependence of the expected cost of the outbreak on the value of *R*_0_ (varied by changing β). Parameter values except where stated in the legends: *N* = 1,000, *R*_0_ = 1.5, μ = 0.1 per day, *I*(0) = 10, *R*(0) = 0, ε = 0.8. For each plot, the average cost is calculated using 100,000 simulations for each value of the number of susceptibles controlled. In D, the plots are of different lengths because there are different numbers of susceptibles available for control for different values of *I*(0). Circles in C, D and E indicate the optimal amount of control to deploy.
(TIF)

S2 Fig Control Amount Optimisation Algorithm (CAOA). Conditional on controlling at the current time, how can the optimal amount of control to deploy be estimated? A. The disease spread parameters are estimated; B. For each possible control amount, the model is simulated forwards many times, and the expected cost of the outbreak is assessed. In each forward simulation, the parameter values are sampled from the posterior in A. In the left panel in B, the blue region is the 95% confidence interval of forwards simulations, with the mean values (dashed black); C. By choosing the amount of control corresponding to the minimum mean forecast cost, the optimal amount of control to deploy is estimated.
(TIF)

S3 Fig Illustration of how the measure of certainty in *R*_0_ is calculated in Fig 1A in the main text. This example corresponds to *t* = 20 days in Fig 1A.
(TIF)

S4 Fig Testing the robustness of our results to informative priors. A. The prior used to simulate underlying datasets (dashed blue) and the prior used to determine the timing and amount of control to use for each strategy (red); B. The distribution of outbreak costs under each control strategy considered; C. The expected cost of outbreaks under each control strategy considered. This analysis is described in more detail in S3 Text.
(TIF)

S5 Fig The CSA outperforms simpler control strategies when multiple parameters require estimation from disease spread data. A. The prior for β used for both the underlying datasets and determining the timing and amount of control to use for each strategy; B. The prior for μ used for both the underlying datasets and determining the timing and amount of control to use for each strategy; C. The resulting effective prior distribution for *R*_0_ calculated by sampling 1,000,000 pairs of values independently out of the priors in A and B; D. The distribution of outbreak costs under each control strategy; E. The expected cost of outbreaks under each control strategy. This analysis is described in more detail in S3 Text.
(TIF)

S6 Fig Extension of the CSA for use in a resource limited setting. A. Schematic illustrating how control is deployed when a decision to control 22 host farms is made, if practical constraints on resource deployment are such that at most 10 host farms can be controlled per day; B. The distribution of outbreak costs under each control strategy; C. The expected cost of outbreaks under each control strategy. This analysis is described in more detail in S3 Text.
(TIF)

S7 Fig Use of the CSA when the prior knowledge is imprecise. A. The priors used for simulating the underlying datasets (dashed blue) and choosing the timing and amount of control to use (red); B. The distribution of outbreak costs under each control strategy; C. The expected cost of outbreaks under each control strategy. In C, black horizontal lines represent the expected cost under each control strategy when the estimation prior is instead equal to the dataset prior, *cf*. S4 Fig, and so quantifies the “cost” of the additional uncertainty in the estimation prior. This analysis is described in more detail in S3 Text.
(TIF)

S8 Fig Performance of the CSA when there is bias in the decision-making, with the policymaker underestimating the transmissibility of the pathogen. A. The priors used for simulating the underlying datasets (blue) and choosing the timing and amount of control to use (red); B. The distribution of outbreak costs under each control strategy; C. The expected cost of outbreaks under each control strategy. This analysis is described in more detail in S3 Text.
(TIF)

S9 Fig Performance of the CSA when there is bias in the decision-making, with the policymaker overestimating the transmissibility of the pathogen. A. The priors used for simulating the underlying datasets (blue) and choosing the timing and amount of control to use (red); B. The distribution of outbreak costs under each control strategy; C. The expected cost of outbreaks under each control strategy. This analysis is described in more detail in S3 Text.
(TIF)

S10 Fig Dynamic programming method for when transmission parameter values are known exactly. Schematic showing how the boundary values of the cost valuation can be used to deduce the cost valuation of each possible state of the SIR model system. Illustration for population size *N* = 11.
(TIF)

S11 Fig Testing that the simulation-based method (equivalent of CSA with known transmission parameters) works when parameter values are known exactly. Policy plots indicate the states of the system in which control should be deployed. A. The amount of control that should be introduced, calculated using dynamic programming; B. Cost valuation, *V*_I,R_, obtained via dynamic programming; C. Policy plot obtained using dynamic programming; D. Policy plot obtained using the simulation-based method with 100,000 simulations per (*I*,*R*) pair, producing similar results to C. Parameter values: *N* = 100, *R*_0_ = 1.5, μ = 0.1 per day, ε = 0.8. The simulation-based method therefore reproduces the same policy plot as the analytical approach, with the advantage that it can be extended to situations in which the parameter values are not known and require estimation (i.e. the CSA).
(TIF)
