# Supplementary material for: Control fast or control smart: When should invading pathogens be controlled?
Source: PLoS Comput Biol. 2018 Feb 16;14(2):e1006014. doi: 10.1371/journal.pcbi.1006014 (PMC5833286; doi:10.1371/journal.pcbi.1006014)
Supplement: S1 Text — (DOCX) [file pcbi.1006014.s001.docx]

**S1. Optimising the amount of control to deploy**

The cost of an outbreak depends in part upon any management interventions that are deployed, and the control strategy to use can be optimised using mathematical models. If control is introduced at a particular time, and the parameters governing disease spread are known exactly, the optimal extent of control to use can be found by scanning over the range of possibilities. In particular, the cost function that we consider is made up of two components, the cost of deploying control and the cost due to farms becoming infected (see Methods). By adding the first of these (S1 Fig A) to the second of these which is approximated using model simulations (S1 Fig B), the cost with each possible amount of control can be found (S1 Fig C). If instead the values of disease transmission parameters are unknown but can be estimated from transmission data, then the forward simulations underlying S1 Fig B are simply replaced by simulations in which the parameter values used are sampled from posterior estimates, giving rise to what we refer to as the Control Amount Optimisation Algorithm (CAOA, S1 Algorithm; S2 Fig).

Deploying control too widely is suboptimal, since too much is then spent on control. However deploying too little control allows the disease to spread leading to a high final size and consequently a high outbreak cost. This sets up a trade-off in which the optimal amount of control to deploy is often an intermediate value (the circles in S1 Fig panels C-E). As shown in S1 Fig panels D and E, the optimal control amount depends both on the numbers of individuals currently infected and also on the parameters governing disease transmission. However, if the size of the outbreak is such that disease fade-out soon is unlikely, and the pathogen is also not extremely widespread in the system, then the optimal control amount is approximately constant (S1 Fig D). For more transmissible pathogens, more control should be deployed (S1 Fig E).
