# Supplementary material for: Control fast or control smart: When should invading pathogens be controlled?
Source: PLoS Comput Biol. 2018 Feb 16;14(2):e1006014. doi: 10.1371/journal.pcbi.1006014 (PMC5833286; doi:10.1371/journal.pcbi.1006014)
Supplement: S2 Text — (DOCX) [file pcbi.1006014.s002.docx]

**S2. Optimising the threshold-based control strategies**

As described in the main text, we compare the expected cost of outbreaks in which control is introduced according to the CSA with those in which control is introduced when more simple criteria are met (Fig 4).

For the threshold-based control strategies, the amount of control to introduce is optimised in advance of the outbreak. For example, in the case of TT, simulations are run with the value of *R*_0_ sampled out of the prior in each simulation. The following is repeated for all possible times of control *t** and number of individuals to control *Q*. If the threshold *t* = *t** is never met, the cost of the single simulated outbreak is simply the number of individuals ever infected during the outbreak, *R*(∞). If the threshold *t* = *t** is met, then *Q* individuals are removed and the outbreak is simulated forwards to obtain a single simulated outbreak cost, *R*(∞) + ε*Q.* This is then repeated for a large number of outbreaks for each possible pairs of values (*t**, *Q*), and the pair corresponding to the lowest expected costs in the simulated outbreaks defines the critical time at which to deploy control, *t*,* as well as the number of individuals to control, *Q*.
