# Supplementary material for: Control fast or control smart: When should invading pathogens be controlled?
Source: PLoS Comput Biol. 2018 Feb 16;14(2):e1006014. doi: 10.1371/journal.pcbi.1006014 (PMC5833286; doi:10.1371/journal.pcbi.1006014)
Supplement: S3 Text — (DOCX) [file pcbi.1006014.s003.docx]

**S3. Elaborations to the Control Smart Algorithm (CSA)**

The CSA can be straightforwardly extended to include complexity omitted from the simple example we showed in the main text (Fig 4). Given posteriors of the values of disease transmission parameters, the CSA integrates over these posteriors and over possible future control strategies to identify the optimal control intervention to perform at the current time. The CSA therefore necessarily outperforms any other strategy based on posteriors for the disease transmission parameters, assuming that these posteriors are an accurate reflection of the values and uncertainties that they represent. Here we demonstrate how elaborations of the CSA can be implemented, and verify that outbreaks with control according to the CSA are on average less costly than those with other control strategies even when such additions to the simple CSA are in place. We note these additional analyses are included to demonstrate that the CSA is robust to a range of complexities, rather than to provide an exhaustive investigation of the magnitude of systematic changes in performance of the CSA with variations in these factors.

In all the elaborations considered here, the value ε = 0.8 is used as is the case in the main text. Except in the case where the value of μ is sampled from a prior distribution (S5 Fig), the value μ = 0.1 per day is used. For these extensions to the CSA, we run simulations in a smaller population of *N* = 100 (for improved computational tractability). This is because in each different case we are testing the performance of the CSA over millions of simulated outbreaks. As commented on in the main text, in practice simulations would only have to be performed on the single outbreak that is ongoing in reality. Simulations would also only have to be run using the single set of priors and only with resource limitations relevant to that single outbreak, significantly reducing computing times compared to the more extensive analyses performed here. Practical use of the CSA is therefore feasible in larger populations and/or with more complex epidemiological models or methods of statistical inference than considered here.

Informed prior knowledge of the parameters

In the main text, we considered the CSA with the values of *R*_0_ used in the underlying datasets picked uniformly at random from the interval (0,5). Estimation in the CSA was also then performed with a prior for *R*_0_ that was uniform on (0,5). Here we instead assume that there is a more informed gamma distributed prior (S4 Fig A). We use gamma distributions with a mode of 1.5 and scale parameter of 0.1. Datasets are generated with *R*_0_ sampled from that distribution, and estimation is performed with that prior. The only change throughout the CSA as described in S2 Algorithm (and by extension in the CAOA) is that each of the posteriors f(*R*_0_|*T*) and f(*R*_0_|*T*+τ) are multiplied by the gamma prior distribution and then renormalised to give a valid probability density function (see S5 Text). The results of the CSA with informative priors are compared to the simpler methods for deciding how to control in S4 Fig B and S4 Fig C. In S4 Fig B – and indeed all the subfigures showing distributions of costs that follow – the widths of bars that are very large are reduced. This permits the rest of the distribution to be observed clearly. For these “large” bars, the percentage chance of an outbreak lying in that bar is given inside it (see e.g. bottom bars on S4 Fig B).

The most important message from this analysis is that more refined prior knowledge of the value of *R*_0_ does not affect our key result: the CSA outperforms the other methods of targeting control.

Estimation of multiple parameters

Here we consider using the CSA when both parameters driving outbreaks in the stochastic SIR model require estimation from data on symptomatic cases. In the case of informative priors described above, the prior for *R*_0_ corresponded to simply a gamma distribution for β, since the value of µ was assumed to be known. Here we instead not only include a gamma distributed prior on the value of β but also a gamma distributed prior on µ (S5 Fig A and B). The resulting effective prior for *R*_0_ can therefore be found by sampling values out of the priors for β and µ, calculating the value of *R*_0_ corresponding to this pair of values, and plotting the resulting distribution of values (S5 Fig C). This effective prior for *R*_0_ is a complicated distribution, since it is the ratio of the two gamma distributions, however we chose parameters for the gamma distributed priors for β and µ so the modes are 0.0015 and 0.1, respectively, which would correspond to a *R*_0_ value of 1.5 = 0.0015**N*/0.1 (i.e. the mode of *R*_0_ considered in the previous example).

In this case, when both parameters must be estimated from observations of symptomatic cases, the only change to the CSA as described in S2 Algorithm (and the CAOA) is that the posterior distributions are no longer simply functions of the single variable *R*_0_. The posteriors f(*R*_0_|*T*) and f(*R*_0_|*T*+τ) are now dependent on both parameters governing transmission, and are therefore instead f($\beta,\mu$|*T*) and f($\beta,\mu$|*T*+τ). Calculation of these posteriors is described below.

The posterior f($\beta,\mu$|*T*) is calculated as follows. First, the likelihood function that is shown in S5 Text is amended to become

$L\left( \beta,\mu\right)=L_{1}\left( \beta,\mu\right) \times L_{2}\left( \beta,\mu\right)$,

where

$L_{1}\left( \beta,\mu\right)=\left( \prod_{i=1}^{n} \boldsymbol{1}_{\mathbf{inf}}\left( t_{i} \right)\beta I\left( t_{i} \right)S\left( t_{i} \right)\exp\left( -\left( \beta I\left( t_{i} \right)S\left( t_{i} \right)+ \mu I\left( t_{i} \right) \right)\left( t_{i}-t_{i-1} \right) \right)+ \left( 1-\boldsymbol{1}_{\mathbf{inf}}\left( t_{i} \right) \right)\mu I\left( t_{i} \right)\exp\left( -\left( \beta I\left( t_{i} \right)S\left( t_{i} \right)+ \mu I\left( t_{i} \right) \right)\left( t_{i}-t_{i-1} \right) \right) \right),$

and

$L_{2}\left( \beta,\mu\right)=\left( \exp\left( -\left( \beta I\left( t_{n+1} \right)S\left( t_{n+1} \right)+ \mu I\left( t_{n+1} \right) \right)\left( t_{n+1}-t_{n} \right) \right) \right)$,

and in which *t*_i_ is the time of the *i*^th^ epidemiological event in the dataset.

Given the observed data up to time *T*, the likelihood surface $L\left( \beta,\mu\right)$ is then constructed by scanning over possible pairs of values of β and µ and calculating $L\left( \beta,\mu\right)$ using these equations. The likelihood surface $L\left( \beta,\mu\right)$ is then multiplied by the each of the priors on β and µ in turn and renormalised to give posterior f($\beta,\mu$|*T*). The posterior f($\beta,\mu$|*T*+τ) is then calculated similarly, given the combination of real and simulated data up to time *T*+τ. When parameters are sampled from the posterior in the CSA, a pair of values ($\beta,\mu$) is found by sampling out of the posterior f($\beta,\mu$|*T*).

The most important message from this analysis is that the need to estimate both individual parameters that make up the value of *R*_0_ – namely $\beta$ and $\mu$ – does not affect our key result: the CSA outperforms the other methods of deciding when and how to intervene.

Logistical constraints in control

We consider an elaboration of the CSA in which limited resources are available for control. This restriction is straightforwardly included in the forward simulations of the CSA. In particular, we consider as an example the case in which it is only possible to deploy vaccination on at most *Y =* 10 farms per day (S6 Fig). In this figure, the priors for *R*_0_ used are gamma distributions with mode and scale parameters of 1.5 and 0.1. A schematic illustrating how vaccination would deployed on 22 farms if control is initiated at time *T* is shown in S6 Fig A. The only difference in the CSA in this case is in step 4, and is built into the CAOA. In the CAOA step 2, when control is deployed on *Q* farms, this control must be staggered. If we denote by *Q*_T_, *Q*_T+1_, *Q*_T+2_,… the number of farms on which to deploy control on days *T*, *T*+1, *T*+2,…, then

$Q_{T}=min(Y,Q)$,

$Q_{T+1}=min(Y,Q-Q_{T})$,

$Q_{T+2}=min(Y,Q-Q_{T+1}-Q_{T})$,

with similar expressions for $Q_{T+3}, Q_{T+4},\ldots$ until vaccination has been deployed on *Q* farms.

The key result of this analysis is that the CSA can be used to assesses how and when to intervene when there are logistic restraints on control deployment. Even in that case, the CSA outperforms simpler methods of deciding when and how to intervene.

Imprecise priors

Until now, to test fully the performance of the CSA over a range of parameter values, we have simulated the datasets using values of the basic reproduction number sampled from a prior. In each case we considered, this prior was also used to inform the different control strategies that we tested. However, in practice the policymaker might only have a very vague idea of the values that the parameters governing disease transmission take compared to the true possible values of these parameters. We therefore consider a situation in which datasets are generated using parameters sampled using from a very tight gamma-distributed prior (mode = 1.5, scale = 0.1), but parameter inference when planning control is then guided by a wider gamma-distributed prior (mode = 1.5, scale = 0.3). Both of these priors are shown in S7 Fig A. As the outbreak progresses, the parameters governing disease spread are learnt with increasing precision, and the CSA once again outperforms the simpler strategies (S7 Fig C).

Bias in decision making

We consider other situations in which the prior used in the CSA is different to the prior used to generate the underlying datasets. In these cases, we consider scenarios in which the policymaker has a prior belief about the value of the basic reproduction number which differs from the prior used to simulated the datasets. In the first example, the policymaker underestimates the transmissibility of the pathogen (S8 Fig): the estimation prior (red) has a mode of 1.5 and scale parameter of 1.3, and the datasets prior (blue) has a mode of 3.5 and scale parameter of 1.3. In the second example, the policymaker overestimates the transmissibility of the pathogen (S9 Fig): the estimation prior (red) has a mode of 2.5 and scale parameter of 1.3, and the datasets prior (blue) has a mode of 1.5 and scale parameter of 1.3.

Waiting before deploying control allows for this bias in decision making to be corrected with evidence about the transmissibility of the pathogen provided by data on symptomatic cases. The performances of the different control strategies are compared in S8 Fig C and S9 Fig C. The key result of these analyses is that, even in these cases in which there is bias in decision making towards incorrect values of *R*_0_, the CSA still outperforms simpler methods of deciding when and how to intervene.
