# Supplementary material for: Control fast or control smart: When should invading pathogens be controlled?
Source: PLoS Comput Biol. 2018 Feb 16;14(2):e1006014. doi: 10.1371/journal.pcbi.1006014 (PMC5833286; doi:10.1371/journal.pcbi.1006014)
Supplement: S4 Text — (DOCX) [file pcbi.1006014.s004.docx]

**S4. Simulations of the stochastic SIR model**

The results in our paper are based on simulations of the stochastic SIR model, in which host units represent individual farms. The variables *S*, *I* and *R* denote the number of susceptible, infected and removed farms, respectively. Outbreaks are modelled as a continuous-time Markov process. The two transitions below are possible and occur at the following rates

| **Event** | **Rate** |
| --- | --- |
| A randomly-chosen susceptible farm becomes infected (S ⇒ I) | β*IS* |
| A randomly-chosen infected farm is removed from the outbreak (I ⇒ R) | μ*I* |

This model is simulated using the direct method version of the Gillespie stochastic simulation algorithm [1-2]. The SIR model and the Gillespie algorithm – as well as other approaches for modelling epidemic processes – are described in detail in the introductory textbook by Keeling and Rohani [3]. Following this, we generate an outbreak simulation as follows:

1. Initialise the number of individuals in each of the *S*, *I* and *R* classes in the model, and set the outbreak time *t* = 0.
2. Steps 2-4 should be repeated while the outbreak is still ongoing (i.e. *I* > 0). First calculate two random numbers r_1_, r_2_ each uniformly distributed in (0,1).
3. Calculate the time of the next event from an exponential distribution. Set

$t=t+ \frac{1}{\beta IS+ \mu I}ln\left( \frac{1}{r_{1}} \right)$.

1. Choose whether the next event is an infection event or removal event. If

$$r_{2}< \frac{\beta IS}{\beta IS+ \mu I},$$

then the next event is an infection event, and so set *S* = *S* – 1 and *I* = *I* + 1. Otherwise set *I* = *I* – 1 and *R = R +* 1.

**References**

1. **Gillespie DT. 1977.** Exact stochastic simulation of coupled chemical reactions. *J Phys Chem* **81**: 2340-2361.
2. **Erban R, Chapman SJ, Maini PK. 2007.** A practical guide to stochastic simulations of reaction-diffusion processes. Available at http://arxiv.org/abs/0704.1908.
3. **Keeling MJ, Rohani P. 2007.** Modeling Infectious Diseases in Humans and Animals. Princeton University Press.
