# Supplementary material for: Control fast or control smart: When should invading pathogens be controlled?
Source: PLoS Comput Biol. 2018 Feb 16;14(2):e1006014. doi: 10.1371/journal.pcbi.1006014 (PMC5833286; doi:10.1371/journal.pcbi.1006014)
Supplement: S5 Text — (DOCX) [file pcbi.1006014.s005.docx]

**S5. Parameter inference**

To inform control decisions, it is necessary to estimate the values of the disease transmission parameters in the farm-level SIR model. Any method of parameter estimation can be integrated into the decision-making framework that we develop. In most of our analyses (except S5 Fig) we assume that the infection rate between farms, *β*, is the only unknown disease transmission parameter, and generate a posterior distribution for the basic reproduction number, *R*_0_. In practice, there is typically more uncertainty in the value of the infection rate than in that of the removal rate, *μ*, since the removal rate can often be estimated from previous epidemics [1] whereas the infection rate depends on the contact rate between individuals, which varies between outbreaks in different settings. We estimate *R*_0_ from the disease transmission data up to time *T*. We assume that the outbreak is observed perfectly - although this is not central to the methodology that we develop - and denote the event times up until time *T* by *t*_1_,*t*_2_,…,*t*_n_. For notational convenience, we also define *t*_0_=0 and *t*_n+1_=*T*. We denote the number of susceptible and infected individuals just before time *t* by *S*(*t*) and *I*(*t*), respectively.

The likelihood function for *R*_0_, in the case where the infection rate is the only unknown parameter value, is given by

$L\left( R_{0} \right)=L_{1}\left( R_{0} \right) \times L_{2}\left( R_{0} \right)$,

where

$L_{1}\left( R_{0} \right)=\prod_{i=1}^{n} \left( \boldsymbol{1}_{\mathbf{inf}}\left( t_{i} \right)\frac{\mu R_{0}}{N}I\left( t_{i} \right)S\left( t_{i} \right)\exp\left( -\left( \frac{\mu R_{0}}{N}I\left( t_{i} \right)S\left( t_{i} \right)+ \mu I\left( t_{i} \right) \right)\left( t_{i}-t_{i-1} \right) \right)+ \left( 1-\boldsymbol{1}_{\mathbf{inf}}\left( t_{i} \right) \right)\mu I\left( t_{i} \right)\exp\left( -\left( \frac{\mu R_{0}}{N}I\left( t_{i} \right)S\left( t_{i} \right)+ \mu I\left( t_{i} \right) \right)\left( t_{i}-t_{i-1} \right) \right) \right)$,

and

$L_{2}\left( R_{0} \right)=\left( \exp\left( -\left( \frac{\mu R_{0}}{N}I\left( t_{n+1} \right)S\left( t_{n+1} \right)+ \mu I\left( t_{n+1} \right) \right)\left( t_{n+1}-t_{n} \right) \right) \right)$.

This form of the likelihood is used extensively (see e.g. O’Neill and Roberts (1999) and Clancy and O’Neill (2008)). The symbol $\boldsymbol{1}_{\mathbf{inf}}\left( t_{i} \right)$ is one if the event at time $t_{i}$ is an infection event, and is zero otherwise. In the stochastic SIR model, the waiting times between events follow an exponential distribution (S4 Text). The first term of the likelihood function, *L*_1_(*R*_0_), is therefore the chance of seeing the observed pattern of infection and removal events up to and including the final event before time *t*_n_. The second term, *L*_2_(*R*_0_), is then the chance of observing the time period between the final infection or removal event and time *t*_n+1_ = *T*, during which there are no events.

In cases in which there is no informative prior for *R*_0_, we renormalise the likelihood function, so that it is a valid probability density function, and use the whole distribution as the posterior estimate for *R*_0_.

In cases in which we include a prior on the value of *R*_0_ in our estimation procedure (i.e. S4-S9 Figs), to find the posterior estimate of *R*_0_ we simply multiply the likelihood function by the prior distribution and renormalise the resulting distribution. This is because the likelihood represents probability of observation the simulation data (***D****,* say) given the parameter value *R*_0_ – i.e. *L*(*R*_0_) = Prob(***D*** | *R*_0_). However, the quantity of interest is instead the posterior distribution – i.e. Prob(*R*_0_ | ***D***). Denoting the prior by Prob(*R*_0_), the posterior for *R*_0_ is related to the prior by Bayes’ rule

$\mathrm{Prob}\left( R_{0} \right|\boldsymbol{D})=\frac{\mathrm{Prob}\left( R_{0}\boldsymbol{\cap D} \right)}{\mathrm{Prob}\left( \boldsymbol{D} \right)}$,

$=\frac{\mathrm{Prob}\left( {\boldsymbol{D}|R}_{0} \right)\mathrm{Prob}\left( R_{0} \right)}{\mathrm{Prob}\left( \boldsymbol{D} \right)}$,

where $\mathrm{Prob}\left( R_{0}\boldsymbol{\cap D} \right)$ is the probability of both the basic reproduction number taking the value *R*_0_ and the data ***D*** being obtained from the simulation. Since the denominator of the final expression, Prob(***D***), is constant for any given dataset, the posterior distribution is simply proportional to the product of the likelihood function and the prior.

**Reference**

1. **Jewell CP, Keeling MJ, Roberts GO.** **2009**. Predicting undetected infections during the 2007 foot-and-mouth disease outbreak. *J R Soc Interface* **41**: 1145-1151.
2. **O’Neill PD, Roberts GO. 1999.** Bayesian inference for partially observed stochastic epidemics. *J R Stat Soc A* **162**: 121-129.
3. **Clancy D, O’Neill PD. 2008.** Bayesian estimation of the basic reproduction number in stochastic epidemic models. *Bayesian Anal* **3**: 737-758.
