# Supplementary material for: Control fast or control smart: When should invading pathogens be controlled?
Source: PLoS Comput Biol. 2018 Feb 16;14(2):e1006014. doi: 10.1371/journal.pcbi.1006014 (PMC5833286; doi:10.1371/journal.pcbi.1006014)
Supplement: S6 Text — (DOCX) [file pcbi.1006014.s006.docx]

**S6. Testing the Control Smart Algorithm (CSA)**

We test the ability of the CSA to identify the optimal time at which to introduce control in a simple setting in which the values of disease spread parameters are assumed to be known exactly. For known parameter values, dynamic programming can be used to find the true optimal time at which to introduce control to minimise the expected cost of simulated outbreaks. We verify that control according to the CSA (in fact, a simplified version of the CSA for known parameter values) is the same as control according to the dynamic programming method.

**Dynamic programming method**

When control can be introduced at any time during an emerging outbreak and the exact values of disease spread parameters are known, the question of whether or not controlling now corresponds to a lower expected outbreak cost than controlling in future can be expressed as a dynamic decision problem and solved using dynamic programming without using stochastic simulation. This approach is based upon Bellman’s Principle of Optimality [1]: *An optimal policy has the property that, whatever the initial action (in the optimal policy), the remaining decisions must constitute an optimal policy with regard to the state resulting from the first decision.* This means that the optimal overall policy is such that any subpolicy is also a set of optimal decisions. Consequently, finding the optimal policy can be broken down into a series of subproblems, leading to a significant reduction in computation time compared to evaluating all possible future control strategies at every single decision time.

We consider the optimal policy to be the one that minimises the expected cost of the outbreak. We define the quantity *V_I,R_* as the minimum expected cost of the outbreak starting from the state (*S,I,R*), assuming that control has not already been deployed and that the optimal control policy is followed in future. We hereafter refer to *V_I,R_* as the “cost valuation” of state (*S,I,R*). Assuming that the policy is followed that minimises the expected cost of the outbreak, then

$$V_{I,R}=\min\left( cost of outbreak if control deployed now, cost of outbreak if control deployed in future \right),$$

$=min\left( \mathbf{E}\left( C_{I,R} \right),\mathbf{E}\left( V_{I_{\mathrm{next}},R_{\mathrm{next}}} \right) \right)$,

where $\mathbf{E}\left( C_{I,R} \right)$ is defined to be the expected cost of the outbreak if control is deployed in the current state (*I,R*), and $V_{I_{\mathrm{next}},R_{\mathrm{next}}}$ is the cost valuation of the state of the system that is visited immediately after the current state (*I,R*). In the SIR model, if the following event is an infection event then (*I*_next_*,R*_next_) = (*I*+1, *R*), and if the following event is a recovery event then (*I*_next_*,R*_next_) = (*I*-1, *R*+1). These two types of event occur with probabilities $\frac{\beta IS}{\beta IS+ \mu I}$ and $\frac{\mu I}{\beta IS+\mu I}$, respectively, where *S* = *N* – *I* – R. Conditioning on the possible values of the next state gives

$$V_{I,R}=\min\left( \mathbf{E}\left( C_{I,R} \right),\sum_{I_{\mathrm{next}},R_{\mathrm{next}}} V_{I_{\mathrm{next}},R_{\mathrm{next}}}\mathbf{P}\left( \left( I_{\mathrm{next}},R_{\mathrm{next}} \right) | \left( I,R \right) \right) \right),$$

which in the specific case of the SIR model is given by

$$V_{I,R}=\min\left( \mathbf{E}\left( C_{I,R} \right), \frac{\beta I\left( N-I-R \right)}{\beta I\left( N-I-R \right)+\mu I}V_{I+1,R}+\frac{\mu I}{\beta I\left( N-I-R \right)+\mu I}V_{I-1,R+1} \right).$$

Noting that *R*_0_ = βN/μ, this can be rewritten as

$$V_{I,R}=\min\left( \mathbf{E}\left( C_{I,R} \right), \frac{R_{0}I\left( N-I-R \right)}{R_{0}I\left( N-I-R \right)+NI}V_{I+1,R}+\frac{NI}{R_{0}I\left( N-I-R \right)+NI}V_{I-1,R+1} \right). (S1)$$

If the values of the population size, *N*, and basic reproduction number, *R*_0_ are known, then the dynamic decision problem (S1) can be solved with appropriate boundary conditions. If, at the current time, all individuals in the system are either infected or removed (i.e. *I*+*R* = *N*), then the cost valuation is known, since the end cost of the epidemic according to the cost metric that we describe in the main text will certainly be *V_I_*_,_*_N_*_-_*_I_* = *N*. If there are currently no infected individuals (i.e. *I* = 0), then the outbreak has finished and the end cost of the epidemic is *V_0_*_,_*_R_* = *R*. The value at every state *V_I_*_,_*_R_* can then be found by solving equation (S1) iteratively using these boundary conditions, working from *R = N* to *R* = 0, as shown in S10 Fig.

Of course, to use this method, it is important to know the values of the $\mathbf{E}\left( C_{I,R} \right)$ terms that appear in equation (S1), i.e. the expected cost of the outbreak if control is deployed in state (*I*,*R*). This can be found by simulating forwards many times with the optimal control amount *Q* found using the CAOA (S1 Algorithm; S2 Fig) in which the input posterior for *R*_0_ consists of the true value of *R*_0_ alone. As well as by simulation, in the specific case of the SIR model the expected cost of an outbreak starting from (*I*,*R*) if *Q* individuals are controlled immediately can also be found by conditioning on the expected cost at neighbouring states, and solving via dynamic programming in the same fashion as calculating *V_I_*_,_*_R_*. Finding $\mathbf{E}\left( C_{I,R} \right)$ then again simply requires searching through *Q* values until the minimum cost is found. For more complicated models, the expected cost if control is deployed in the state (*I*,*R*), i.e. $\mathbf{E}\left( C_{I,R} \right)$, can be found by simulation.

Control should then be applied in any state (*I*,*R*) where the expected cost of outbreaks with control at the current time is lower than the expected cost valuation of the following state, i.e. any state in which

$\mathbf{E}\left( C_{I,R} \right)< \mathbf{E}\left( V_{I_{\mathrm{next}},R_{\mathrm{next}}} \right)$**.**

**Simulation-based method (known parameters)**

We propose a simulation-based method that can be used to decide whether to control at the current time, or instead wait until the next possible control time (shown for possible control times τ = 0,1,2,... in S3 Algorithm, although emendation for other possible times of control is straightforward). This method is identical to the CSA in the simplified case in which the values of the parameters governing disease spread are known exactly. The expected cost of the outbreak if control is deployed now and the equivalent cost if control is applied in the optimal way in future are compared. The results of this algorithm are equivalent to those of the dynamic decision problem (S1) in simple scenarios in which the system is observed continually and the values of disease spread parameters are known. We illustrate this using “policy plots” in S11 Fig. The benefit of this simulation-based approach is that it can be extended to more complex cases, such as when disease transmission parameters require estimation, leading to the CSA (see main text Fig 2 and S2 Algorithm).

**Reference**

1. **Bellman R. 1954.** The theory of dynamic programming. *Bull Am Math Soc* **60**: 503– 516.
