# Supplementary material for: Control fast or control smart: When should invading pathogens be controlled?
Source: PLoS Comput Biol. 2018 Feb 16;14(2):e1006014. doi: 10.1371/journal.pcbi.1006014 (PMC5833286; doi:10.1371/journal.pcbi.1006014)
Supplement: S1 Algorithm — (DOCX) [file pcbi.1006014.s007.docx]

**Algorithm S1.** Control Amount Optimisation Algorithm (CAOA).

1. Use the transmission data up to the current time, *T*, to estimate the posterior estimate of *R*_0_, which we denote by f(*R*_0_ | *T*).
   1. For *Q =* 1,2,…,*S*(*T*), run a large number *M* simulations forwards from time *T*, applying control to *Q* individuals. In each simulation, use parameter values sampled from the posterior f(*R*_0_ | *T*). Evaluate the expected cost of outbreaks in which *Q* individuals are controlled, *C*_Q_.
2. Find the value of *Q* for which *C*_Q_ is minimised: call this *Q**, say. Control *Q** individuals.
