# Supplementary material for: Control fast or control smart: When should invading pathogens be controlled?
Source: PLoS Comput Biol. 2018 Feb 16;14(2):e1006014. doi: 10.1371/journal.pcbi.1006014 (PMC5833286; doi:10.1371/journal.pcbi.1006014)
Supplement: S2 Algorithm — (DOCX) [file pcbi.1006014.s008.docx]

**Algorithm S2.** Control Smart Algorithm (CSA): for deciding whether or not to control at the current time while estimating parameters from disease spread data.

1. Using data from the outbreak up to the current time, *T*, estimate the values of disease transmission parameters, giving posterior f(*R*_0_|*T*). This is the only time that real outbreak data are used in the algorithm.
2. Make a single sample from the posterior f(*R*_0_|*T*): suppose that the particular sampled parameters are *R*_0_*.
3. Simulate forwards from time *T* to time *T*+τ, using parameters *R*_0_*. Update the posterior estimate of the parameters during this simulation, giving a simulated (fake) future posterior estimate f(*R*_0_|*T*+τ).
4. Use the updated posterior, f(*R*_0_|*T*+τ), to calculate the amount of control that would be deployed in the simulation at time *T*+τ, using the CAOA (S1 Algorithm). In the simulation, deploy this amount of control and continue simulating forwards using parameters *R*_0_* until the outbreak has ended. This gives the cost of a simulated outbreak, conditional on controlling at time *T*+τ.
5. Repeat the algorithm from step 2, *M* times for each of the possible times which control decisions can be made, e.g. τ = 0,1,2,…. Consequently construct the expected cost of the outbreak conditional on control at time *T*+τ, for each of τ = 0,1,2,…, which we denote by *C*_T_(τ).
6. If *C*_T_(0) < min_τ_*_=_*_1,2,3,…_(*C*_T_(τ)), then, on average, the current time is the best time at which to control. In that case, deploy control now according to the CAOA (S1 Algorithm), and denote the current time *T* = *T**. Otherwise wait until the next possible control time, and restart this algorithm.
