# Supplementary material for: Control fast or control smart: When should invading pathogens be controlled?
Source: PLoS Comput Biol. 2018 Feb 16;14(2):e1006014. doi: 10.1371/journal.pcbi.1006014 (PMC5833286; doi:10.1371/journal.pcbi.1006014)
Supplement: S3 Algorithm — (DOCX) [file pcbi.1006014.s009.docx]

**Algorithm S3.** Simulation-based method for deciding whether or not to control at the current time (analogous to the CSA but with parameters known).

1. Observe the system at the current time, *T*. Set τ = 0.
2. Run *M* simulations forwards to time T+τ*.*
3. In each simulation: determine the optimal amount of control to deploy at time T+τ using the CAOA (but with the posterior for *R*_0_ consisting of the true value of *R*_0_ alone), and implement this amount of control.
4. Continue the simulations forwards until the epidemic has ended, and obtain the expected cost of outbreaks with control at time T+τ, which we denote as *C*_T_(τ).
5. Set τ *=* τ*+*1. Repeat from step 2 until τ is sufficiently large that all simulated outbreaks have ended (i.e. *I*(T+τ) = 0 in any forward simulation).
6. If *C*_T_(0) < min_τ_*_=_*_1,2,3,…_(*C*_T_(τ)), then control now. Otherwise wait until the following decision time.
