# Supplementary figures and images for: Control fast or control smart: When should invading pathogens be controlled?
Source: PLoS Comput Biol. 2018 Feb 16;14(2):e1006014. doi: 10.1371/journal.pcbi.1006014 (PMC5833286; doi:10.1371/journal.pcbi.1006014)

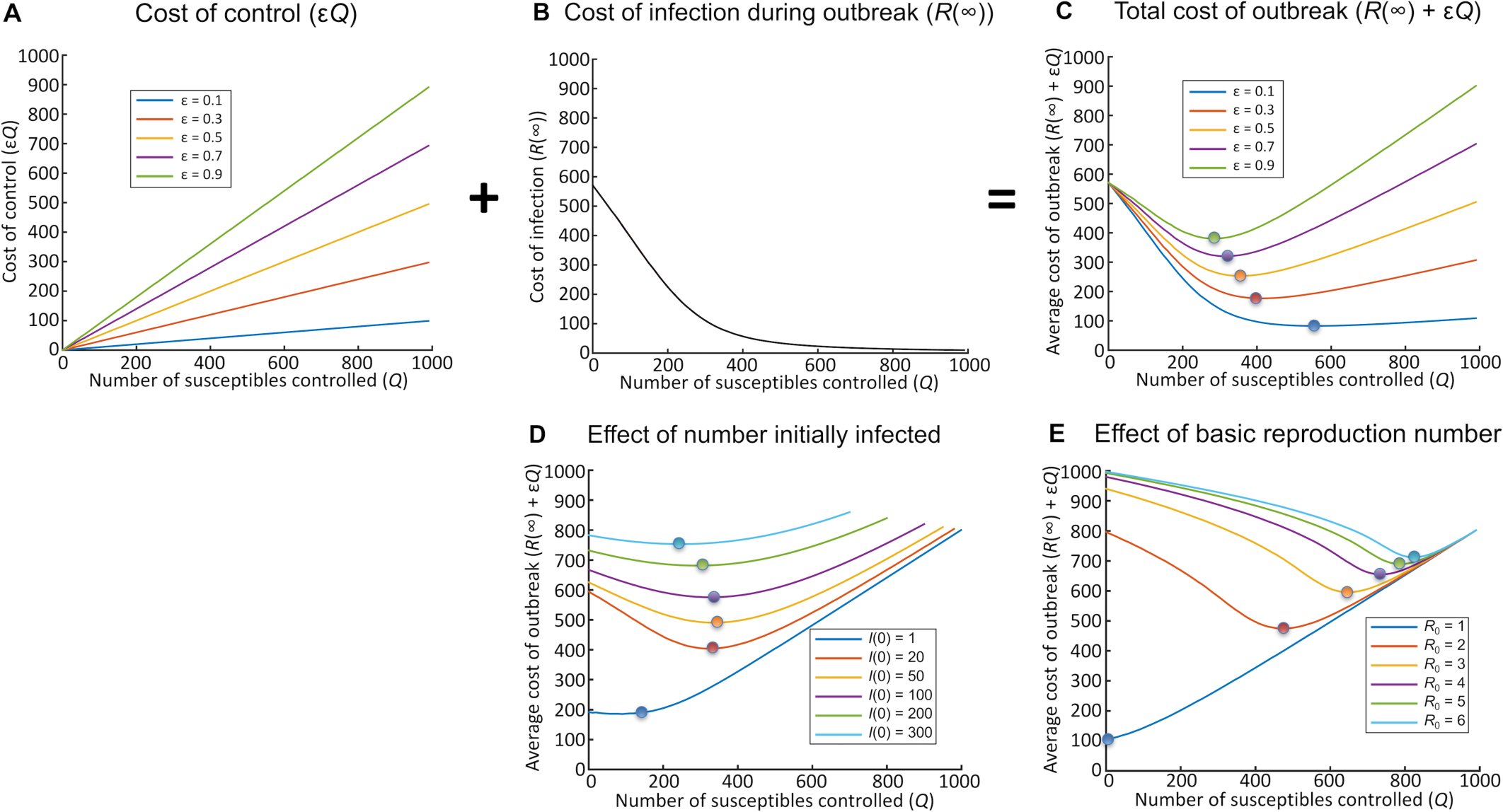

Supplement: S1 Fig — A. Cost due to control, εQ, for different costs of control ε; B. Expected cost due to individuals becoming infected during the outbreak, R(∞); C. Expected total cost of the outbreak for different costs of control ε, evaluated as the sum of A and B; D. Dependence of the expected cost of the outbreak on different initial numbers of infected individuals; E. Dependence of the expected cost of the outbreak on the value of R0 (varied by changing β). Parameter values except where stated in the legends: N = 1,000, R0 = 1.5, μ = 0.1 per day, I(0) = 10, R(0) = 0, ε = 0.8. For each plot, the average cost is calculated using 100,000 simulations for each value of the number of susceptibles controlled. In D, the plots are of different lengths because there are different numbers of susceptibles available for control for different values of I(0). Circles in C, D and E indicate the optimal amount of control to deploy. (TIF) [file pcbi.1006014.s010.tif]

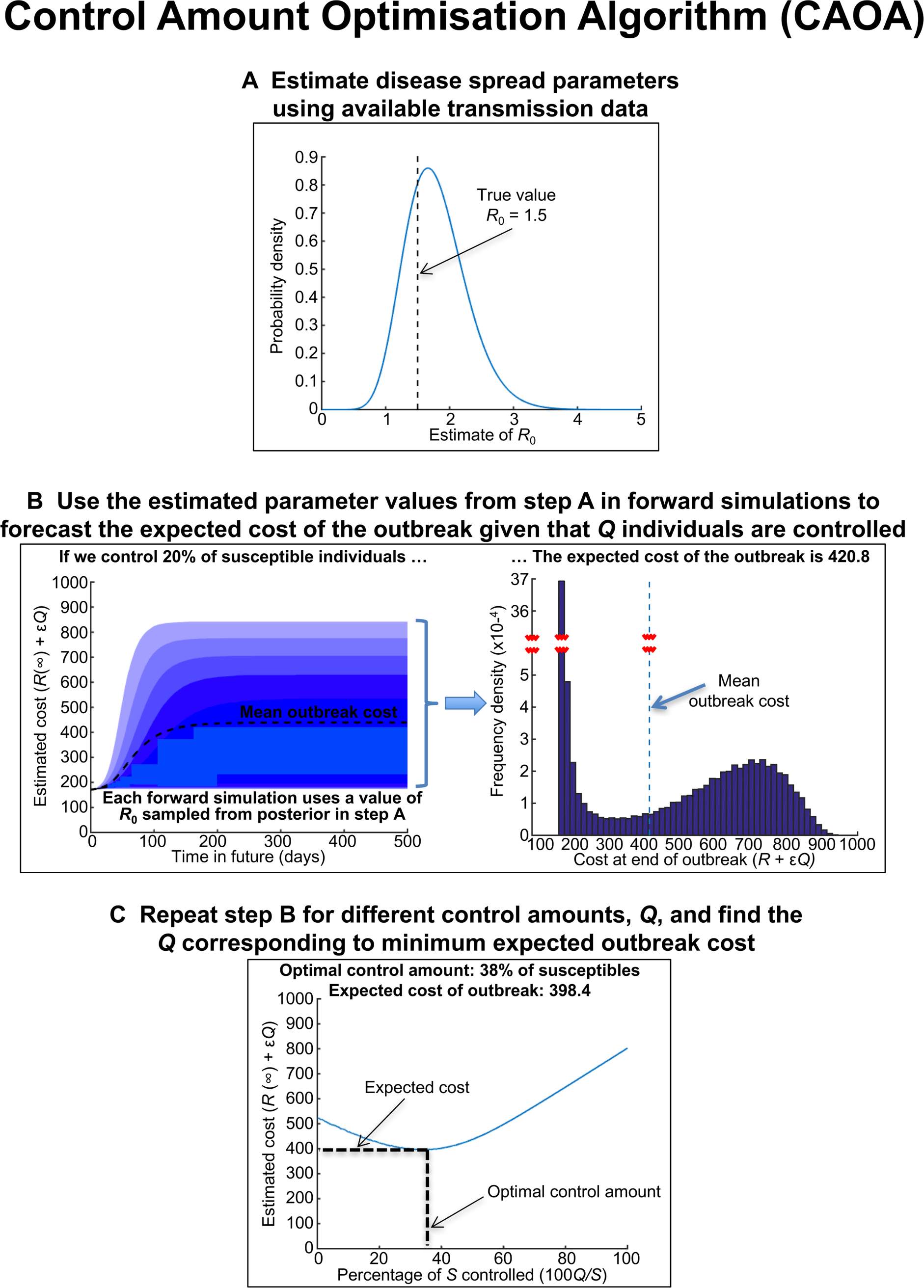

Supplement: S2 Fig — Conditional on controlling at the current time, how can the optimal amount of control to deploy be estimated? A. The disease spread parameters are estimated; B. For each possible control amount, the model is simulated forwards many times, and the expected cost of the outbreak is assessed. In each forward simulation, the parameter values are sampled from the posterior in A. In the left panel in B, the blue region is the 95% confidence interval of forwards simulations, with the mean values (dashed black); C. By choosing the amount of control corresponding to the minimum mean forecast cost, the optimal amount of control to deploy is estimated. (TIF) [file pcbi.1006014.s011.tif]

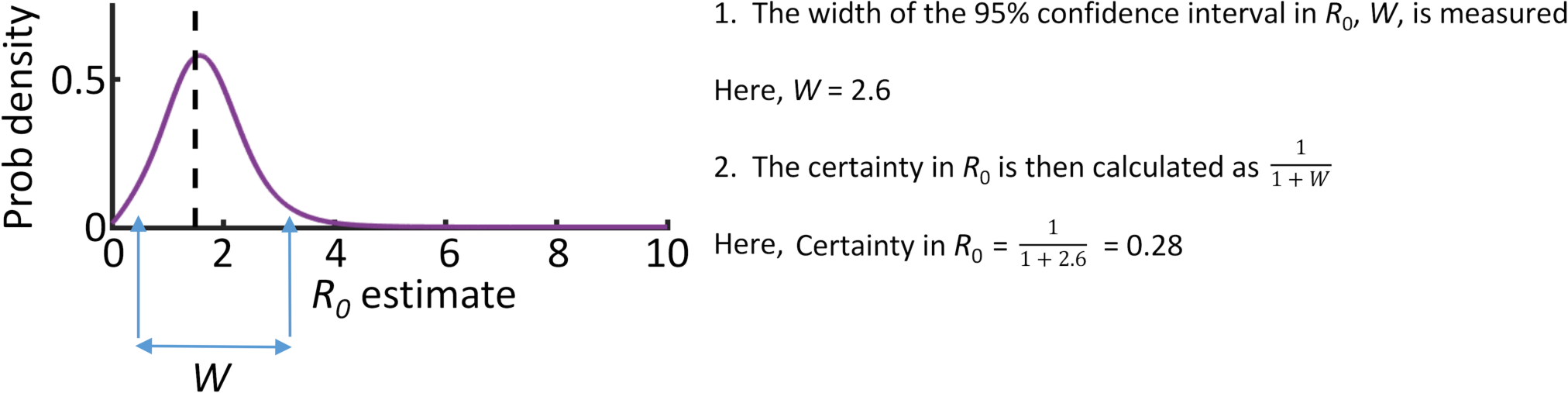

Supplement: S3 Fig — This example corresponds to t = 20 days in Fig 1A. (TIF) [file pcbi.1006014.s012.tif]

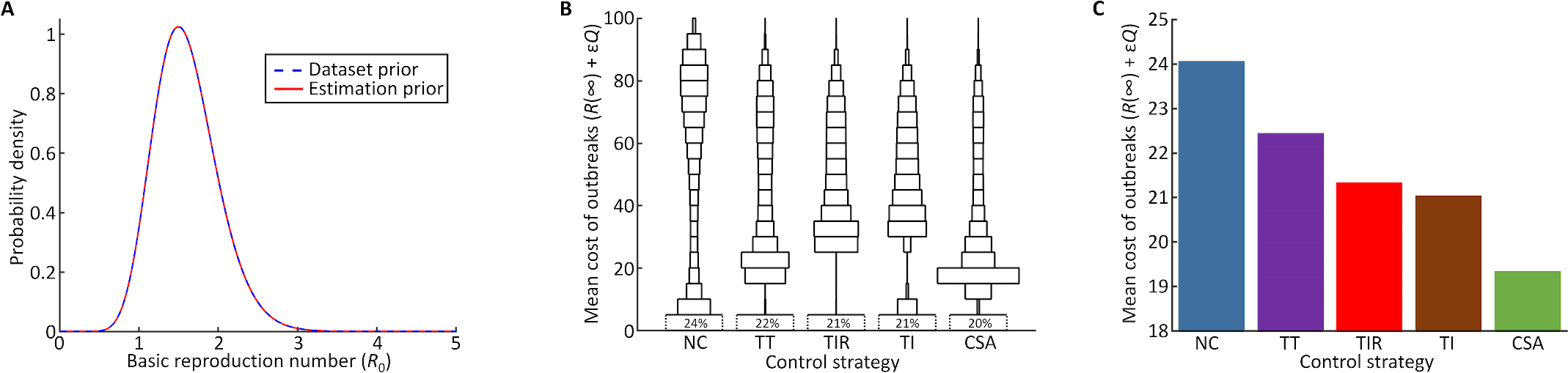

Supplement: S4 Fig — A. The prior used to simulate underlying datasets (dashed blue) and the prior used to determine the timing and amount of control to use for each strategy (red); B. The distribution of outbreak costs under each control strategy considered; C. The expected cost of outbreaks under each control strategy considered. This analysis is described in more detail in S3 Text. (TIF) [file pcbi.1006014.s013.tif]

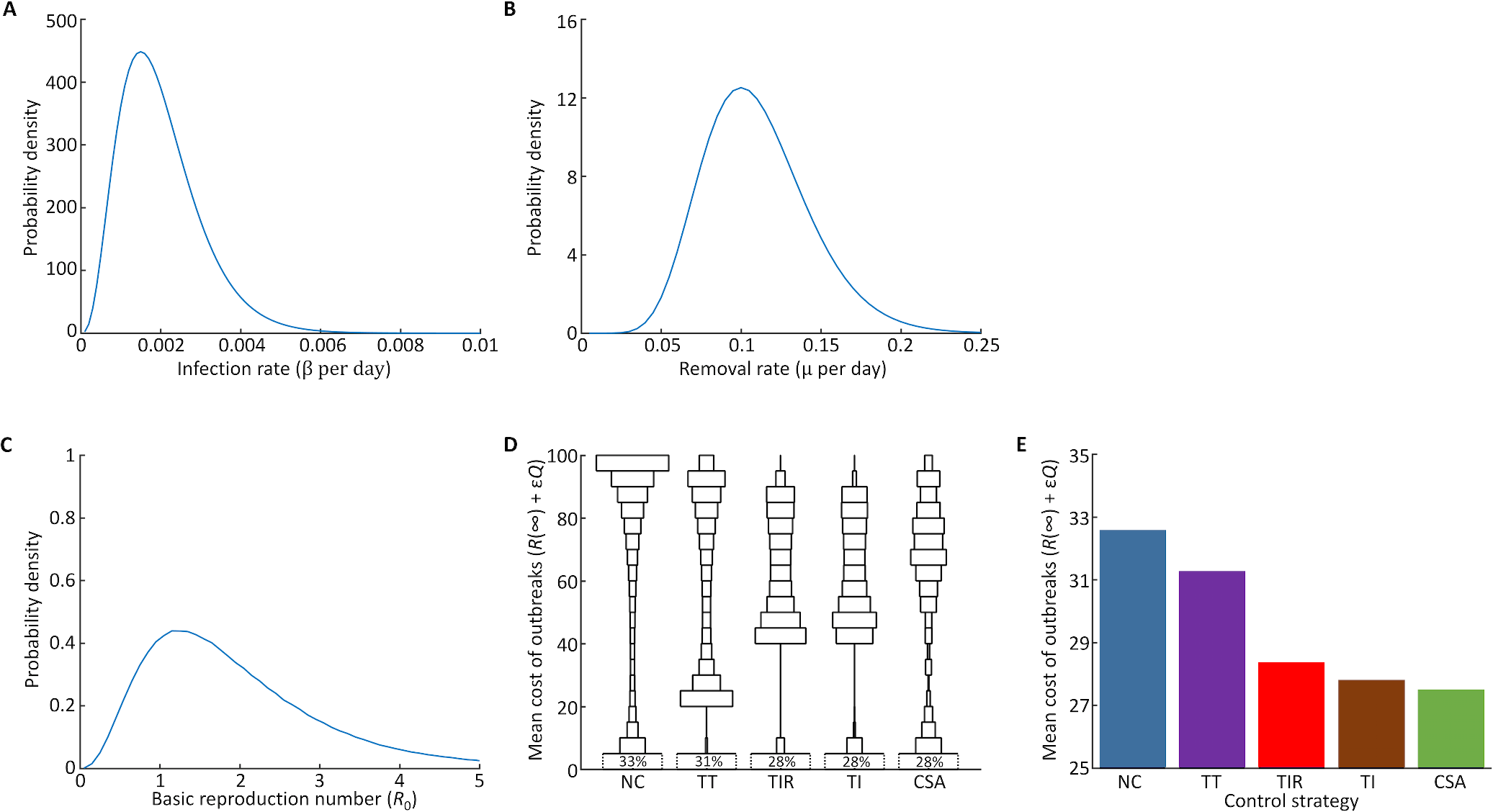

Supplement: S5 Fig — A. The prior for β used for both the underlying datasets and determining the timing and amount of control to use for each strategy; B. The prior for μ used for both the underlying datasets and determining the timing and amount of control to use for each strategy; C. The resulting effective prior distribution for R0 calculated by sampling 1,000,000 pairs of values independently out of the priors in A and B; D. The distribution of outbreak costs under each control strategy; E. The expected cost of outbreaks under each control strategy. This analysis is described in more detail in S3 Text. (TIF) [file pcbi.1006014.s014.tif]

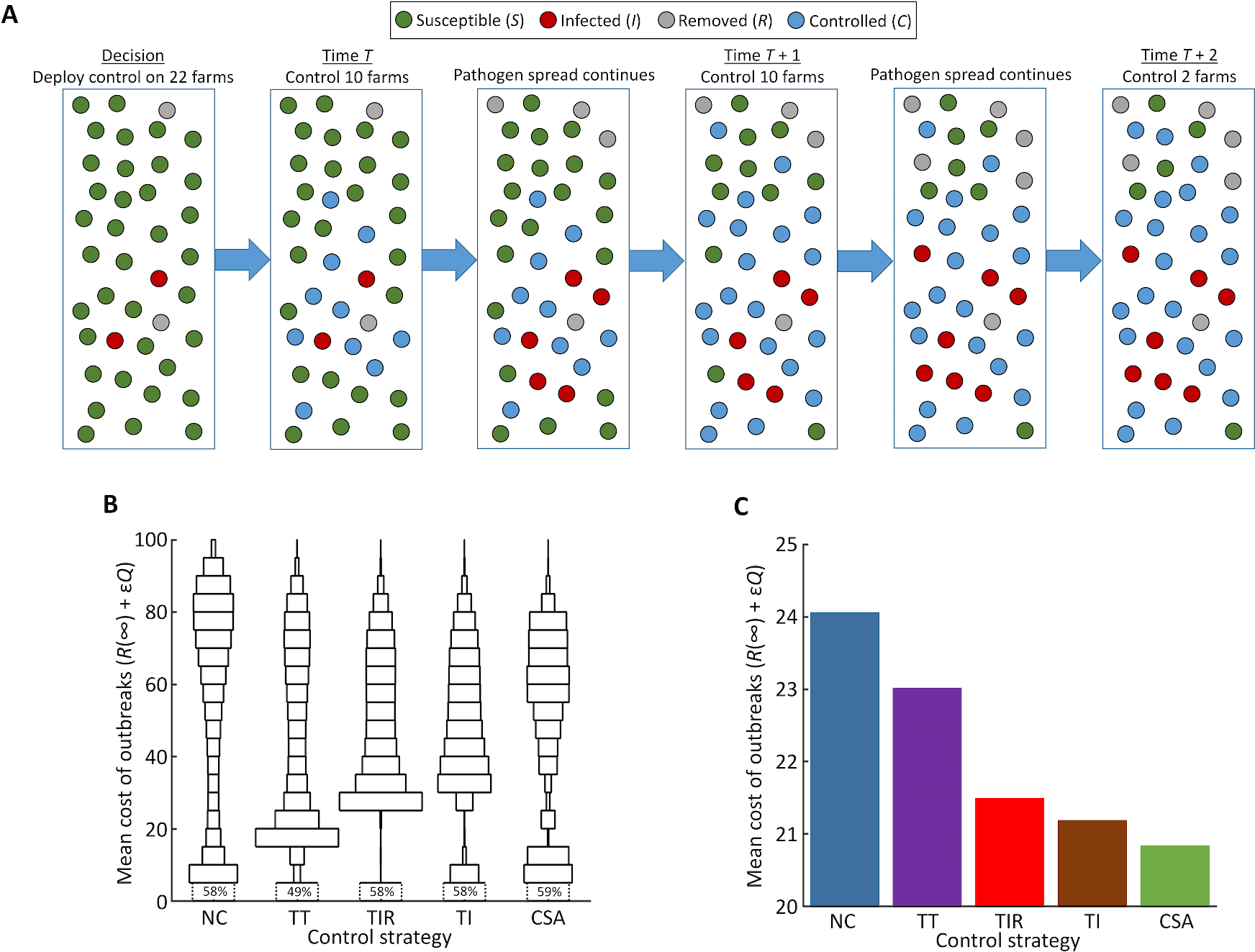

Supplement: S6 Fig — A. Schematic illustrating how control is deployed when a decision to control 22 host farms is made, if practical constraints on resource deployment are such that at most 10 host farms can be controlled per day; B. The distribution of outbreak costs under each control strategy; C. The expected cost of outbreaks under each control strategy. This analysis is described in more detail in S3 Text. (TIF) [file pcbi.1006014.s015.tif]

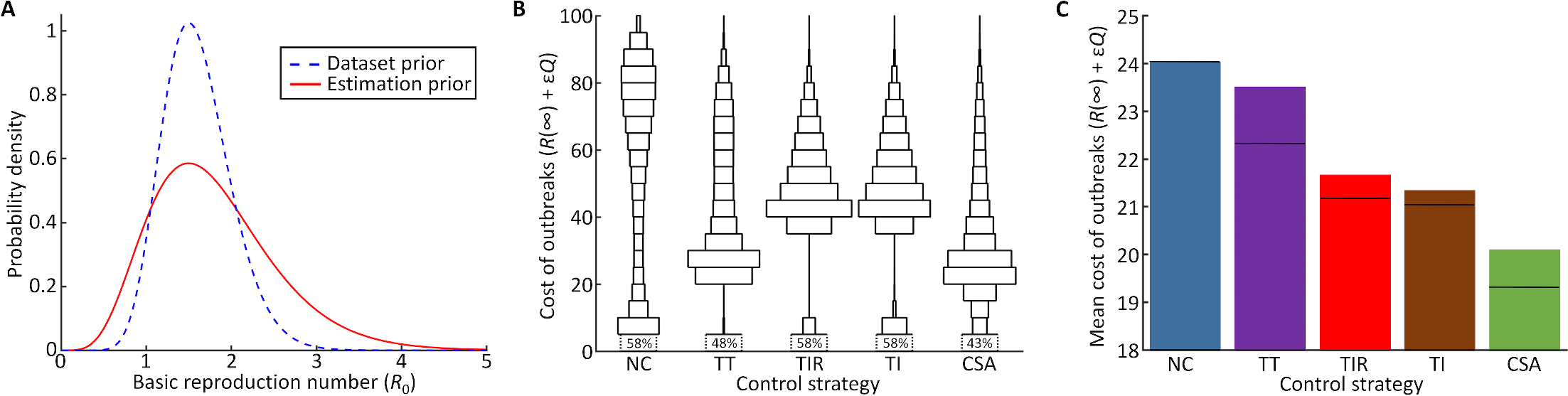

Supplement: S7 Fig — A. The priors used for simulating the underlying datasets (dashed blue) and choosing the timing and amount of control to use (red); B. The distribution of outbreak costs under each control strategy; C. The expected cost of outbreaks under each control strategy. In C, black horizontal lines represent the expected cost under each control strategy when the estimation prior is instead equal to the dataset prior, cf. S4 Fig, and so quantifies the “cost” of the additional uncertainty in the estimation prior. This analysis is described in more detail in S3 Text. (TIF) [file pcbi.1006014.s016.tif]

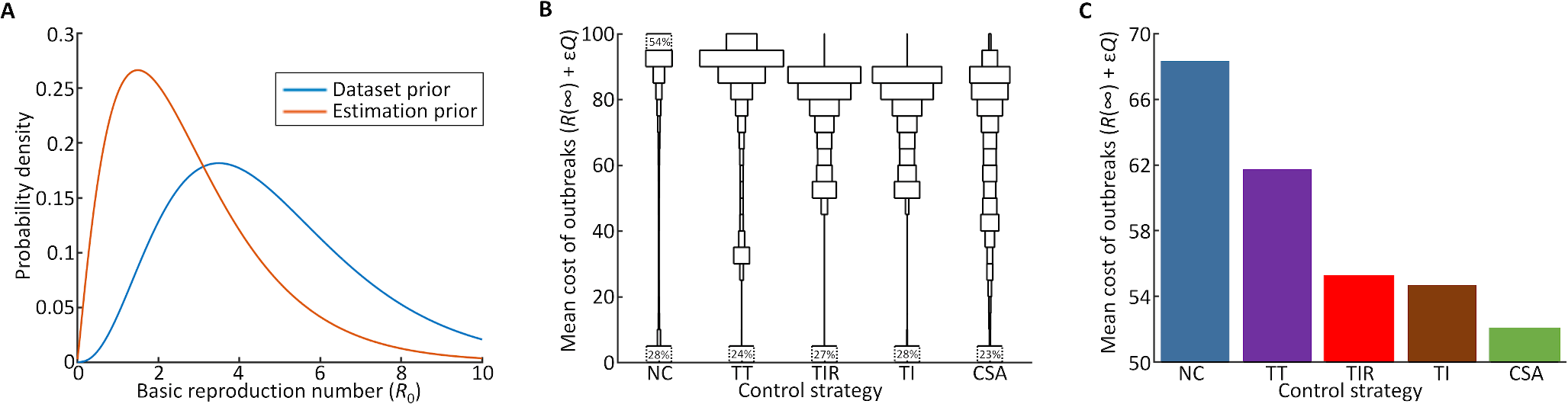

Supplement: S8 Fig — A. The priors used for simulating the underlying datasets (blue) and choosing the timing and amount of control to use (red); B. The distribution of outbreak costs under each control strategy; C. The expected cost of outbreaks under each control strategy. This analysis is described in more detail in S3 Text. (TIF) [file pcbi.1006014.s017.tif]

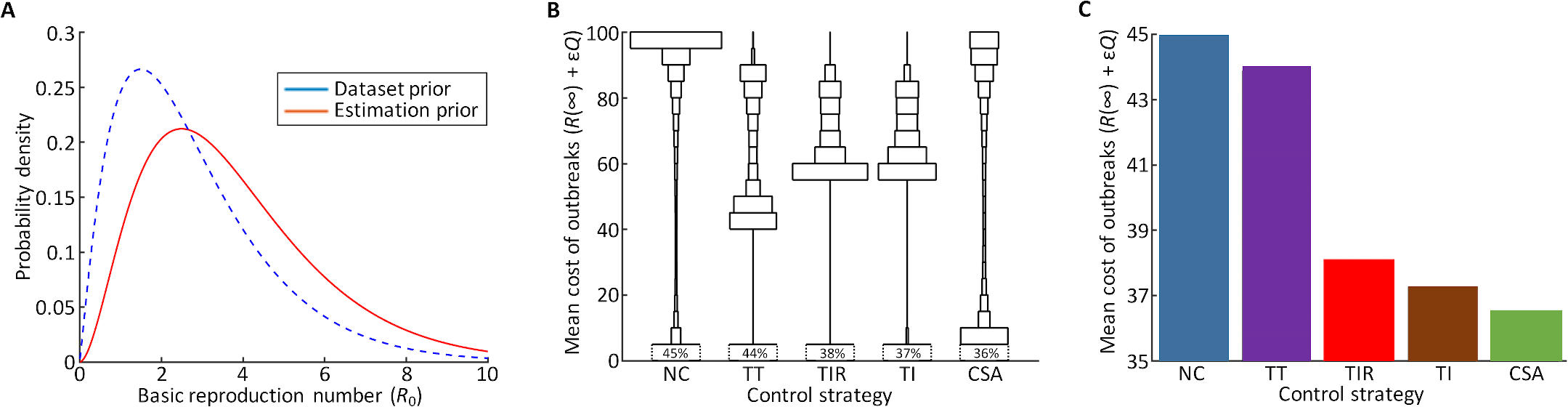

Supplement: S9 Fig — A. The priors used for simulating the underlying datasets (blue) and choosing the timing and amount of control to use (red); B. The distribution of outbreak costs under each control strategy; C. The expected cost of outbreaks under each control strategy. This analysis is described in more detail in S3 Text. (TIF) [file pcbi.1006014.s018.tif]

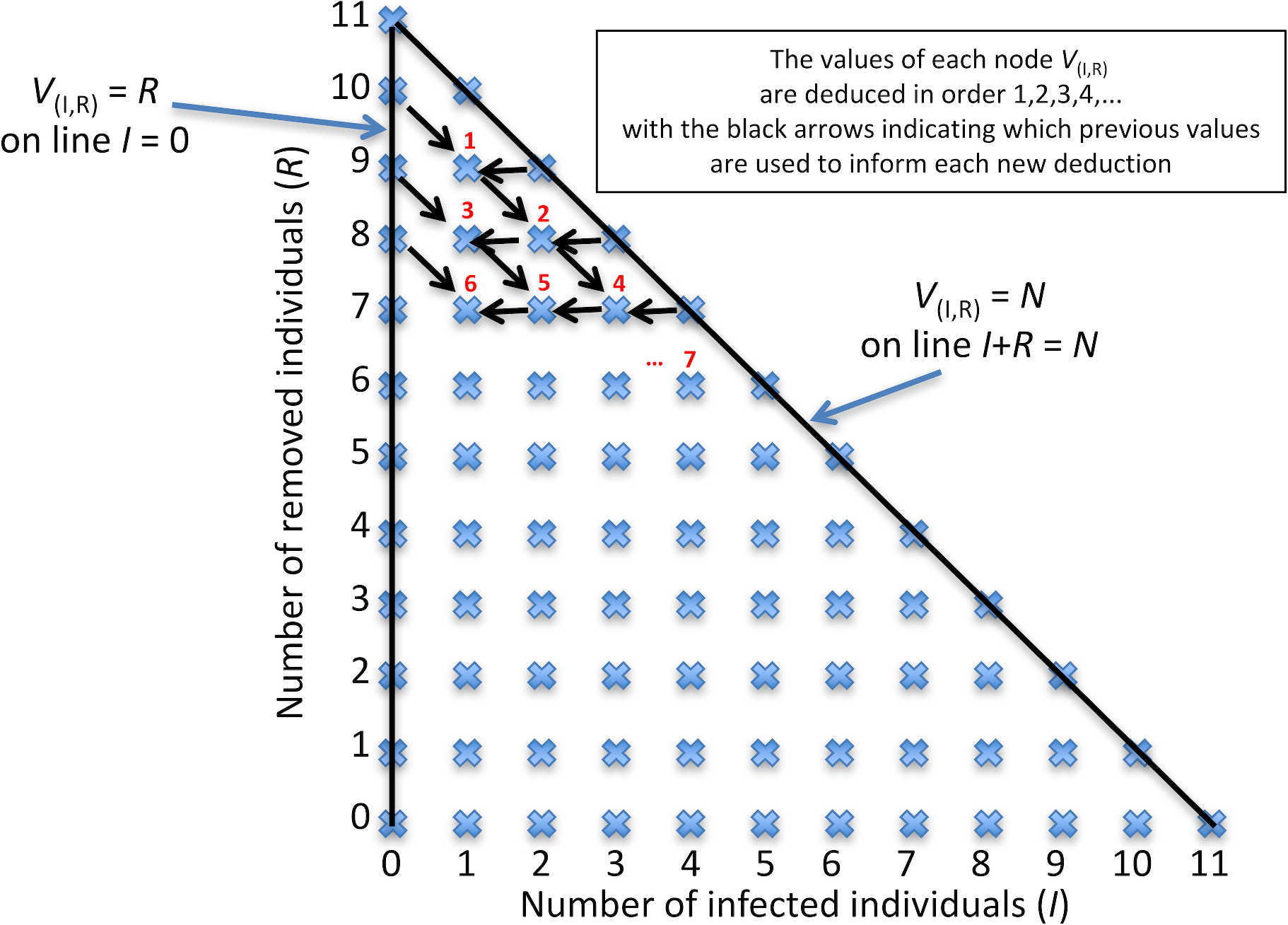

Supplement: S10 Fig — Schematic showing how the boundary values of the cost valuation can be used to deduce the cost valuation of each possible state of the SIR model system. Illustration for population size N = 11. (TIF) [file pcbi.1006014.s019.tif]

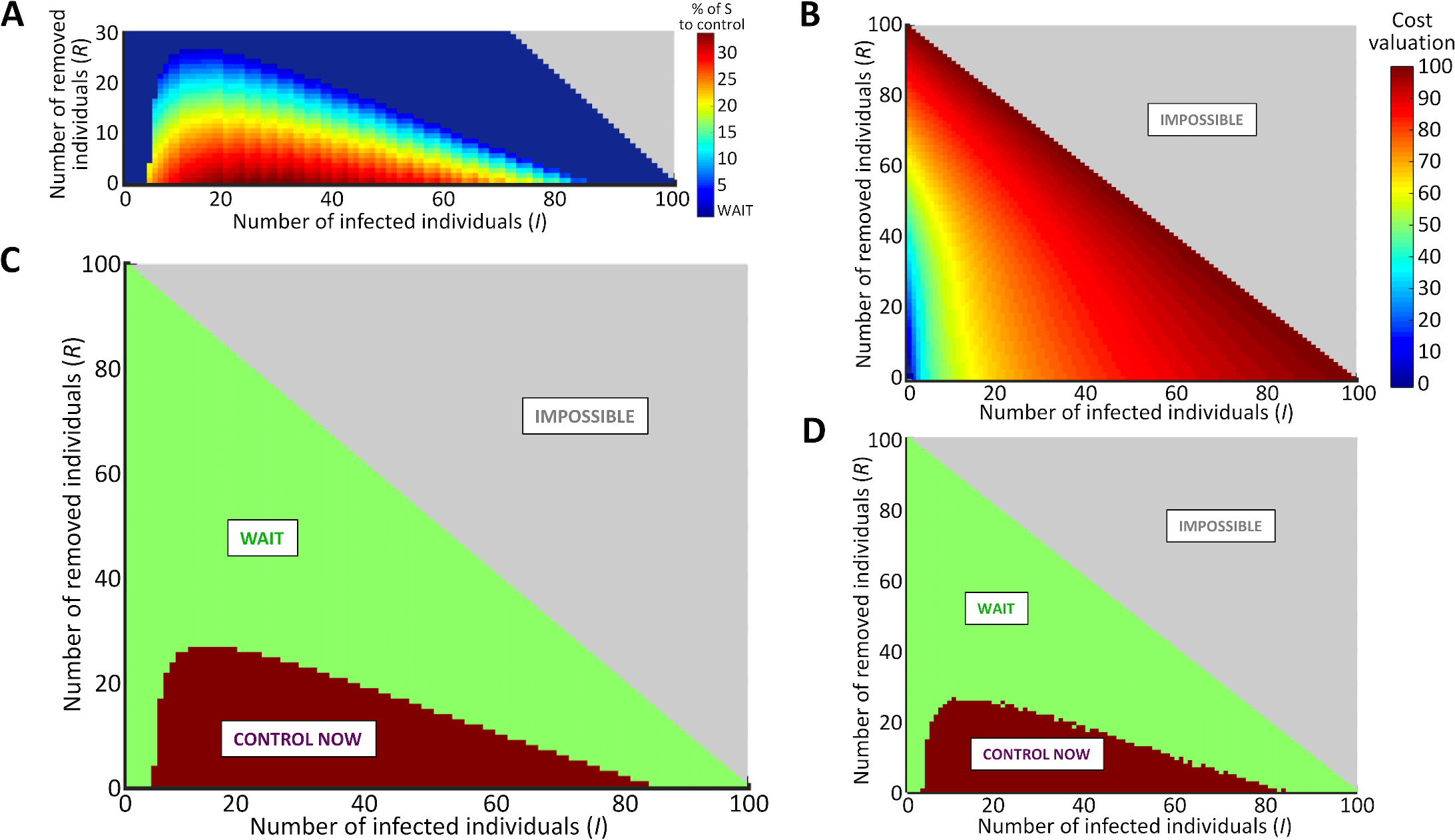

Supplement: S11 Fig — Policy plots indicate the states of the system in which control should be deployed. A. The amount of control that should be introduced, calculated using dynamic programming; B. Cost valuation, VI,R, obtained via dynamic programming; C. Policy plot obtained using dynamic programming; D. Policy plot obtained using the simulation-based method with 100,000 simulations per (I,R) pair, producing similar results to C. Parameter values: N = 100, R0 = 1.5, μ = 0.1 per day, ε = 0.8. The simulation-based method therefore reproduces the same policy plot as the analytical approach, with the advantage that it can be extended to situations in which the parameter values are not known and require estimation (i.e. the CSA). (TIF) [file pcbi.1006014.s020.tif]
